# Plasma and Fecal Metabolites Combined with Gut Microbiome Reveal Systemic Metabolic Shifts in ^60^Co Gamma-Irradiated Rats

**DOI:** 10.3390/metabo15060363

**Published:** 2025-05-29

**Authors:** Jie Zong, Haiyang Wu, Xuan Hu, Ami Yao, Wenhua Zhu, Guifang Dou, Shuchen Liu, Xiaoxia Zhu, Ruolan Gu, Yunbo Sun, Zhuona Wu, Shanshan Wang, Hui Gan

**Affiliations:** Beijing Institute of Radiation Medicine, Beijing 100850, China; zong_jie0929@163.com (J.Z.); ldsxwhy@126.com (H.W.); xuan347883203@163.com (X.H.); 15055736910@163.com (A.Y.); 15866981562@163.com (W.Z.); 13681022512@163.com (X.Z.); guruolan@bmi.ac.cn (R.G.); sunyunbo0919@126.com (Y.S.); wznphd@126.com (Z.W.); 15562317238@163.com (S.W.)

**Keywords:** radiation, ^60^Coγ-ray, untarget metabolomics, 16S rRNA sequencing, gut microbiota, metabolic disruption

## Abstract

**Background**: High-dose γ-ray exposure (≥7 Gy) in nuclear emergencies induces life-threatening acute radiation syndrome, characterized by rapid hematopoietic collapse (leukocytes <0.5 × 10⁹/L) and gastrointestinal barrier failure. While clinical biomarkers like leukocyte depletion guide current therapies targeting myelosuppression, the concomitant metabolic disturbances and gut microbiota dysbiosis—critical determinants of delayed mortality—remain insufficiently profiled across the 28-day injury-recovery continuum. **Methods**: This study investigates the effects of ^60^Co γ-ray irradiation on metabolic characteristics and gut microbiota in Sprague Dawley rats using untargeted metabolomics and 16S rRNA sequencing. Meanwhile, body weight and complete blood counts were measured. **Results**: Body weight exhibited significant fluctuations, with the most pronounced deviation observed at 14 days. Blood counts revealed a rapid decline in white blood cells, red blood cells, and platelets post-irradiation, reaching nadirs at 7–14 days, followed by gradual recovery to near-normal levels by 28 days. Untargeted metabolomics identified 32 upregulated and 33 downregulated plasma metabolites at 14 days post-irradiation, while fecal metabolites showed 47 upregulated and 18 downregulated species at 3 days. Key metabolic pathways impacted included Glycerophospholipid metabolism, alpha-linolenic acid metabolism, and biosynthesis of unsaturated fatty acids. Gut microbiota analysis demonstrated no significant change in α-diversity but significant *β*-diversity shifts (*p* < 0.05), indicating a marked alteration in the compositional structure of the intestinal microbial community following radiation exposure. Principal coordinate analysis confirmed distinct clustering between control and irradiated groups, with increased abundance of *Bacteroidota* and decreased *Firmicutes* in irradiated rats. These findings highlight dynamic metabolic and microbial disruptions post-irradiation, with recovery patterns suggesting a 28-day restoration cycle. Spearman’s rank correlation analysis explored associations between the top 20 fecal metabolites and 50 abundant bacterial taxa. *Norank_f_Muribaculaceae*, *Prevotellaceae_UCG-001*, and *Bacteroides* showed significant correlations with various radiation-altered metabolites, highlighting metabolite–microbiota relationships post-radiation. **Conclusions**: This study provides insights into potential biomarkers for radiation-induced physiological damage and underscores the interplay between systemic metabolism and gut microbiota in radiation response.

## 1. Introduction

Ionizing radiation (IR) serves as a pivotal environmental and clinical mediator, exerting profound biological consequences that encompass acute tissue injury to chronic metabolic dysregulation [1,2]. Among diverse radiation modalities, ^60^Co γ-ray irradiation remains extensively employed in radiation oncology and radiobiology research, a preference attributed to its deep-tissue penetration capacity and precise dose calibration capabilities [3]. To investigate metabolic reprogramming during radiation recovery, we established a rat model of acute radiation injury. A total-body irradiation dose of 7 Gy is recognized as the definitive threshold for hematopoietic acute radiation syndrome (h-ARS) in animal models, consistently inducing severe bone marrow suppression and persistent leukopenia within 7 days post-exposure [4,5]. While these hematologic perturbations are hallmark manifestations of h-ARS [6], the systemic metabolic changes and their interactions with gut microbiota during recovery remain poorly understood. Recent developments in omics technologies, particularly untargeted metabolomics and 16S rRNA sequencing, provide powerful tools to investigate these complex biological relationships [7,8].

Previous studies on non-human primates have indicated that carnitine metabolites play important roles in radiation-related metabolic regulation. These metabolites are potentially involved in fatty acid metabolism [9,10,11]. Meanwhile, tricarboxylic acid (TCA) cycle intermediates (e.g., citrate, succinate) were perturbed in plasma and urine, consistent with radiation-induced mitochondrial dysfunction [12,13,14]. These findings align with amino acid pathway dysregulation observed across gastrointestinal, urinary, and serum compartments, highlighting multi-tissue metabolic reprogramming post-irradiation [15]. These findings, combined with perturbations in oxidative stress markers (e.g., depleted glutathione, a master antioxidant; elevated malondialdehyde, reflecting lipid peroxidation) [16,17,18] and gut microbiota-derived metabolites (e.g., reduced short-chain fatty acids critical for intestinal barrier integrity; altered indole derivatives linked to microbial tryptophan metabolism) [19,20], underscore systemic metabolic remodeling post-irradiation. The gastrointestinal microbiome undergoes profound restructuring following radiation exposure, exhibiting distinct alterations across multiple biological tiers. Compositionally, radiation exposure disrupts the homeostatic *Firmicutes/Bacteroidota* balance through selective phylum-level shifts [21,22]. Functionally, this dysbiotic state correlates with impaired short-chain fatty acid biosynthesis and redirected tryptophan metabolic flux—perturbations mechanistically associated with compromised intestinal barrier integrity and immune–endocrine crosstalk disruption [23,24]. However, most research has focused on acute-phase responses (<7 days post-irradiation), which creates a knowledge gap in understanding both metabolic recovery dynamics and their association with gut microbial resilience. Furthermore, although certain microbial metabolites (e.g., short-chain fatty acids) have demonstrated potential to alleviate radiation damage in animal studies [20], current datasets remain fragmented across experimental models. Large-scale profiling of microbiota–metabolite associations is urgently required to map consistent interaction patterns and identify high-priority targets for radiation countermeasure development.

To address these research gaps, this study uses a longitudinal approach to track metabolic and gut microbial changes over time in Sprague-Dawley rats exposed to 7 Gy ^60^Co γ-ray radiation. By combining plasma and feces metabolomic analysis with 16S rRNA sequencing of gut bacteria, we focus on three key aspects: identifying critical periods of metabolic disruption and recovery, mapping radiation-specific changes in major metabolic pathways, and exploring connections between gut microbial communities and host metabolism. Our results reveal new insights into how organisms adapt to radiation exposure and suggest possible treatment targets for radiation-related metabolic problems.

## 2. Materials and Methods

### 2.1. Chemicals and Reagents

High-purity chromatographic-grade solvents, including water, methanol, and formic acid (Thermo Fisher Scientific, Santa Clara, CA, USA; Catalog NO. W6-4, A456-4, A117-50), were employed in this investigation. All chemical reagents demonstrated chromatographic purity levels exceeding 99% as certified by the manufacturer.

### 2.2. Animals and Experimental Design

Sixteen male Sprague Dawley rats (SPF grade, 160–180 g) were procured from Charles River Laboratories (Beijing, China, certificate No. SCXK [Beijing] 2021-0040) and acclimatized in controlled barrier facilities. The vivarium maintained standardized environmental parameters with a 12-h photoperiod, ambient temperature of 20–25 °C, and relative humidity at 40–70%. Our experimental schemes and all surgical and experimental procedures were endorsed and approved by the Beijing Institute of Radiation Medicine (Beijing, China, IACUC-DWZX-2024-P626). Operational workflows strictly complied with the 3R framework to minimize biological stress, supported by real-time monitoring of behavioral indicators to ensure animal welfare compliance.

Sixteen SD rats were randomly divided into the control group (A group) and irradiation group (B group), with each group containing 8 rats. After a 3-day acclimatization period under controlled conditions, irradiation group received 7 Gy whole-body γ-irradiation using a cobalt-60 source, while control groups underwent sham procedures without radiation.

### 2.3. Sample Collection, Complete Blood Count, and Pretreatment

Blood samples were collected from the orbital vein using a centrifuge tube containing EDTA, followed by centrifugation at 3500× *g* for 15 min to obtain plasma the day before (Blank) and 6 h, 1 day, 3 days, 7 days, 14 days, 21 days, and 28 days after irradiation. Complete blood counts (CBC) were analyzed using a Mindray BC-5000Vet hematology analyzer. Feces were collected at matched time points by placing rats in a sterilized biosafety cabinet and inducing defecation through gentle perianal stimulation. Fresh fecal pellets were transferred into cryovials, snap-frozen in liquid nitrogen, and stored at −80 °C. According to the requirements of sample preparation, 100.0 μL of plasma samples (n = 8 per group) were collected for metabolomics analysis. Plasma metabolites were extracted by vortexing in 300 μL of cold methanol, followed by centrifugation at 12,000× *g* for 10 min. After centrifugation, 300 μL of supernatant was collected and dried using a vacuum centrifuge. The dried sample was then reconstituted by adding 100 μL of 50% methanol followed by sonication. For fecal metabolite extraction, lyophilized specimens (50 mg) were homogenized with 1 mL of 80% (*v*/*v*) methanol aqueous solution. After centrifugation (12,000× *g*, 10 min), 200 μL of the resulting supernatant was subjected to metabolomic profiling.

### 2.4. Untargeted Metabolomics Assay of Plasma Metabolites

Untargeted metabolomic profiling was performed using a Dionex UltiMate 3000-Q Exactive system (Thermo Scientific. Ltd., Waltham, MA, USA). A Waters UPLC HSS T3 column (1.8 μm, 2.1 mm × 100 mm, Waters Corporation, Milford, MA, USA) was used to separate metabolites. The mobile phase consisted of (A) 0.1% formic acid in water and (B) methanol (100%), with the following gradient program: initial conditions were maintained at 98% A for 1 min, followed by a linear decrease to 0% A over 5.5 min. This composition was held for 14 min before returning to 98% A within 0.1 min, with final equilibration for 2 min (total run time 16 min). Metabolites were identified based on exact masses within 5 ppm and standard retention times. Relative metabolite levels were quantified using chromatographic peak areas, with abundance normalized to fecal sample weight or plasma volume. Multivariate statistical analysis and pathway enrichment were conducted using SIMCA (version 14.1, Umetrics Umeå, Sweden) and MetaboAnalyst 5.0, respectively.

### 2.5. DNA Sequencing and Gut Microbiota Analysis

Total genomic DNA was extracted from fecal samples using the FastPure Stool DNA Isolation Kit (MJYH, Shanghai, China) according to the manufacturer’s protocols. DNA integrity was verified through 1.0% agarose gel electrophoresis, while concentration and purity were quantified via NanoDrop2000 spectrophotometry (Thermo Scientific. Ltd., Waltham, MA, USA). Equimolar amplicon pools were sequenced on an Illumina Nextseq2000 PE3000 platform (Illumina. Inc., San Diego, CA, USA) following Majorbio Bio-Pharm Technology Co. Ltd. (Shanghai, China). Demultiplexed raw reads were processed through fastp (v0.19.6) [25] for quality control and FLASH (v1.2.11) [26] for read merging. Sequence denoising was implemented via the DADA2 algorithm [27] in QIIME2 (v2020.2) [28] with default parameters, generating amplicon sequence variants (ASVs) through sample-specific error modeling to achieve single-nucleotide resolution. Taxonomic assignment of ASVs was carried out by applying the Naive Bayes consensus taxonomy classifier integrated into Qiime2 and the SILVA 16S rRNA database (v138.2). The obtained results were then analyzed via the Majorbio cloud platform (https://www.MajorBio.com, accessed on 15 March 2025).

### 2.6. Statistical Analyses

Physiological data are expressed as mean ± SD. Graphical representations were generated with GraphPad Prism 8.0.1. Multivariate analyses, including principal component analysis (PCA) and orthogonal partial least squares-discriminant analysis (OPLS-DA), were performed using SIMCA (version 14.1, Umetrics) for metabolic pattern recognition, with Benjamini–Hochberg false discovery rate correction (FDR α = 0.05) applied to metabolomics data [29]. Principal coordinate analysis (PCoA) was conducted via the MicrobiomeAnalyst platform to evaluate intergroup variations. Spearman’s rank correlation analysis was subsequently applied to determine associations between gut microbiota alterations and differential metabolite profiles across experimental groups.

## 3. Results

### 3.1. Body Weights and Complete Blood Counts

**Figure 1:**
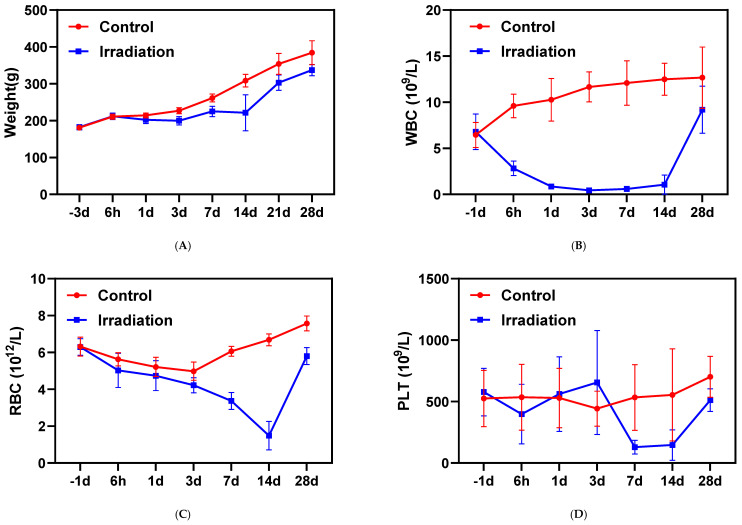
Longitudinal changes in body weight and hematological parameters of rats following ^60^Co γ-irradiation: (**A**) Body weight measured 3 days before radiation and up to 28 days post-exposure; (**B**) White blood cell (WBC) counts 1 days before radiation and up to 28 days post-exposure; (**C**) Red blood cell (RBC) counts 1 days before radiation and up to 28 days post-exposure; (**D**) Platelet (PLT) counts 1 days before radiation and up to 28 days post-exposure.

Body weight showed dynamic changes post-irradiation, with an initial decline (days 1–3), transient recovery (days 3–7), and marked inter-individual variation peaking at day 14. These patterns suggest days 1–14 represent the critical recovery window, with inter-individual differences peaking at day 14. Subsequently, weight rebounded substantially from day 14 to 28, approaching control levels (Figure 1A). Whole-body irradiation triggered a rapid decline in white and red blood cell counts, reaching their lowest levels at days 3–7 and day 14 (Figure 1B,C). This pattern indicates hematopoietic damage, with complete recovery occurring within 28 days, suggesting a full regeneration cycle spanning approximately one month. Although platelet counts showed substantial variability due to their wide physiological range in normal rats (400–1600 × 10⁹/L), the values still reached minimum levels between days 7–14 post-treatment (Figure 1D).

### 3.2. Temporal Hotspot of Radiation-Induced Metabolic Shifts

**Figure 2:**
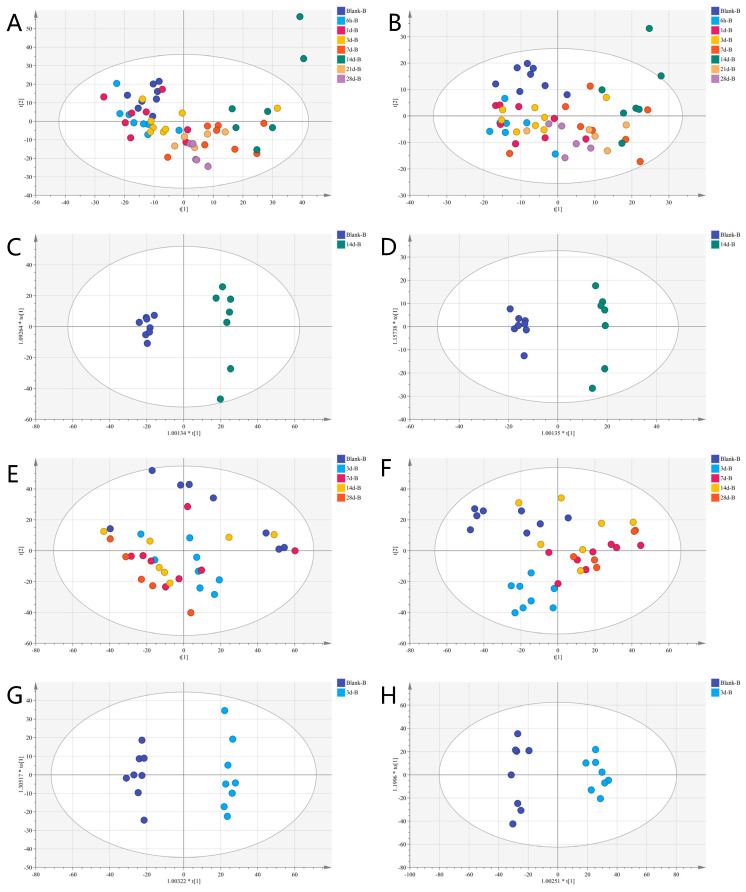
PCA and PCoA plots of plasma and fecal metabolomes across time points: (**A**) PCA of plasma metabolomes (positive ionization mode) reveals time-dependent clustering; (**B**) PCA of plasma metabolomes (negative ionization mode) reveals time-dependent clustering; (**C**) PCoA of plasma metabolites in positive ionization mode at 14 days post-intervention (R^2^Y = 0.985, Q^2^ = 0.941); (**D**) PCoA of plasma metabolites in negative ionization mode at 14 days post-intervention (R^2^Y = 0.988, Q^2^ = 0.942); (**E**) PCA of fecal metabolome (positive ionization mode) reveals time-dependent clustering; (**F**) PCA of fecal metabolome (negative ionization mode) reveals time-dependent clustering; (**G**) PCoA of fecal metabolome in positive ionization mode at 3 days post-intervention (R^2^Y = 0.989, Q^2^ = 0.813); (**H**) PCoA of fecal metabolome in negative ionization mode at 3 days post-intervention (R^2^Y = 0.989, Q^2^ = 0.934). The asterisk (*) denotes multiplication, scaling the component score (t[1]) for optimal visualization.

To assess the metabolic changes induced by ionizing radiation in rats, we used the Dionex UltiMate 3000-Q Exactive system for untargeted metabolome profiling of the rats’ plasma samples. The principal component analysis (PCA) model revealed that the metabolites in the Blank-B group (the day before IR), 6 h-B group (6 h after IR), 1d-B group (1 day after IR), 3d-B group (3 days after IR), 7 d-B group (7 days after IR), 14 d-B group (14 days after IR), 21 d-B group (21 days after IR), and 28 d-B group (28 days after IR) were clearly separated. Figure 2A,B shows that from the 6h-B group to the 14 d-B group, groups gradually moved away from the Blank-B group, and the 21 d-B and 28 d-B groups gradually moved closer to the Blank-B group. In particular, the difference was greatest between the 14 d-B group and the Blank-B group (Figure 2A,B). We then performed Orthogonal Partial Least Squares Discriminant Analysis (OPLS-DA) on the Blank-B and 14 d-B groups, and the results indicated that the metabolic composition of the 14 d-B group was completely separated from that of the Blank-B group (Figure 2C,D). Untargeted metabolomics profiling of fecal samples from the Blank-B, 3 d-B, 7 d-B, 14 d-B, and 28 d-B groups demonstrated a clear shift from the Blank-B group starting on day 3, with this distinction persisting throughout the 28-day observation period (Figure 2E,F). We then performed OPLS-DA analysis on the Blank-B and 3 d-B groups, and the results still showed that the metabolic composition of the 3 d-B group was completely separated from that of the Blank-B group (Figure 2G,H).

### 3.3. Metabolomics Analysis

**Figure 3:**
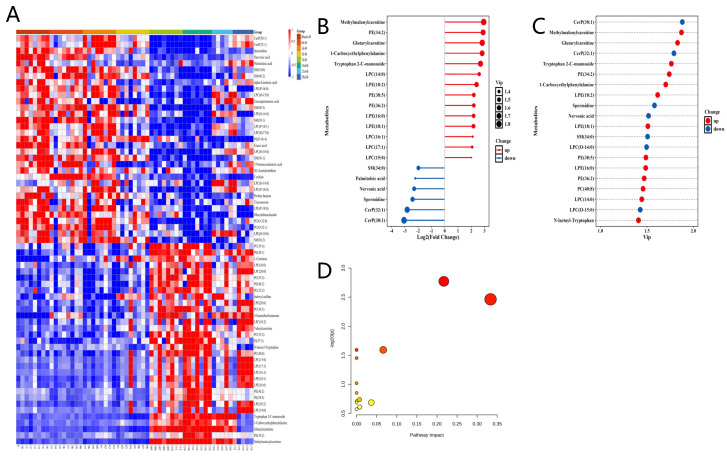
Radiation-induced alterations in plasma metabolites at 14 days post-exposure: (**A**) Heatmap visualization of differentially abundant metabolites across experimental groups; (**B**) Rank-ordered metabolite alterations: top 20 candidates by fold change magnitude; (**C**) VIP-prioritized discriminatory metabolites: top 20 candidates ranked by Variable Importance in Projection (VIP) scores; (**D**) Significantly enriched KEGG pathways visualized by bubble plot. Red hues indicate increased abundance; blue hues indicate decreased abundance.

**Figure 4:**
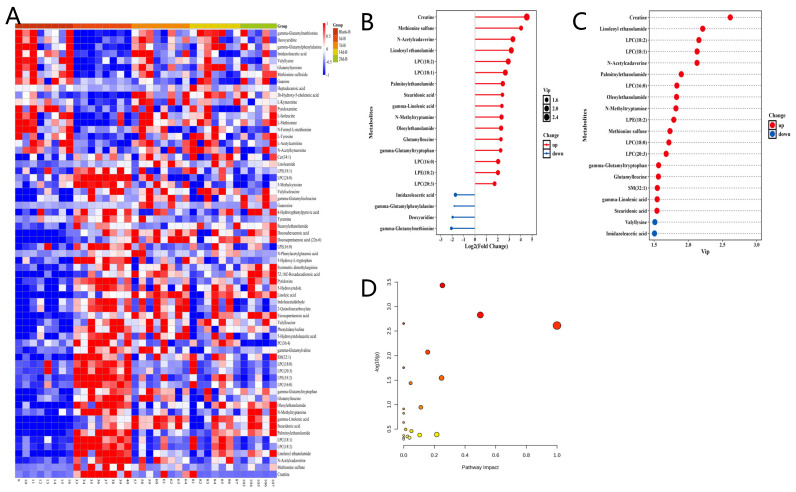
Radiation-induced alterations in fecal metabolites at 3 days post-exposure: (**A**) Heatmap visualization of differentially abundant metabolites across experimental groups; (**B**) Rank-ordered metabolite alterations: top 20 candidates by fold change magnitude; (**C**) VIP-prioritized discriminatory metabolites: top 20 candidates ranked by Variable Importance in Projection (VIP) scores; (**D**) Significantly enriched KEGG pathways visualized by bubble plot. Red hues indicate increased abundance; blue hues indicate decreased abundance.

Compared to the plasma Blank-B group, the 14 d-B group exhibited significant changes in metabolite abundance, with 32 metabolites showing increased levels and 33 metabolites displaying reduced levels (Figure 3A). In contrast, when analyzing fecal samples, the 3 d-B group demonstrated more pronounced alterations: 47 metabolites were upregulated and 18 metabolites were downregulated relative to the feces Blank-B group. These differentially abundant metabolites were further visualized in a hierarchical clustering heatmap (Figure 4A). Figure 3B,C illustrates the key metabolites responsible for distinguishing plasma groups, as identified by orthogonal partial least squares-discriminant analysis (OPLS-DA), with metabolite selection criteria of fold change > 2.0 and variable importance in projection (VIP) scores > 1.0. Similarly, Figure 4B,C presents the analysis of characteristic fecal metabolites selected under identical criteria, demonstrating their distinct roles in group discrimination. The main differential metabolites in plasma include the following: methylmalonylcarnitine; phosphatidylethanolamine (PE) (34:2); glutarylcarnitine; 1-carboxyethylphenylalanine; tryptophan 2-C-mannoside; lysophosphatidylcholine (LPC) (14:0); lysophosphatidylethanolamine (LPE) (18:2); PE(38:5); PE(36:2); LPE(16:0); LPE(18:1); LPC(16:1); LPC(17:1); LPC(15:0); SM(34:0); palmitoleic acid; nervonic acid; spermidine; ceramide phosphate (CerP) (30:1); CerP(32:1); LPC (O-14:0); phosphatidylcholine (PC) (40:8); LPC(O-15:0); and N-lactoyl-tryptophan (Figure 3B,C). The temporal dynamics of these compounds are illustrated in Supplementary Figures S1 and S2. The main differential metabolites in feces include the following: creatine; methionine sulfone; N-acetylcadaverine; linoleoyl ethanolamide; LPC(18:2); LPC(18:1); palmitoylethanolamide; stearidonic acid; gamma-Linolenic acid; N-methyltryptamine; oleoylethanolamide; glutamylleucine; gamma-glutamyltryptophan; LPC(16:0); LPE (18:2); LPC(20:3); imidazoleacetic acid; gamma-glutamylphenylalanine; deoxyuridine; gamma-glutamylmethionine; LPC(18:0); sphingomyelin (SM) (32:1); and valyllysine (Figure 4B,C). The temporal dynamics of these compounds are illustrated in Supplementary Figures S3 and S4. The variations of these compounds over time are depicted in the accompanying figures. Pathway enrichment analysis of the KEGG database revealed that IR treatment significantly modulated key metabolic pathways in plasma, including the following: glycerophospholipid metabolism, alpha-linolenic acid metabolism, biosynthesis of unsaturated fatty acids, arginine and proline metabolism, linoleic acid metabolism, arginine biosynthesis, beta-alanine metabolism, glutathione metabolism, and other related pathways (Table 1). Notably, IR treatment significantly perturbed fecal metabolic pathways, including the following: tryptophan metabolism, phenylalanine, tyrosine and tryptophan biosynthesis, biosynthesis of unsaturated fatty acids, linoleic acid metabolism, vitamin B6 metabolism, alpha-linolenic acid metabolism, tyrosine metabolism, and related pathways (Table 2). Common metabolic pathways in both plasma and feces included the following: glycerophospholipid metabolism, alpha-linolenic acid metabolism, biosynthesis of unsaturated fatty acids, linoleic acid metabolism, arginine and proline metabolism, sphingolipid metabolism, pyrimidine metabolism, and arachidonic acid metabolism.

### 3.4. Microbiota Analysis

**Figure 5:**
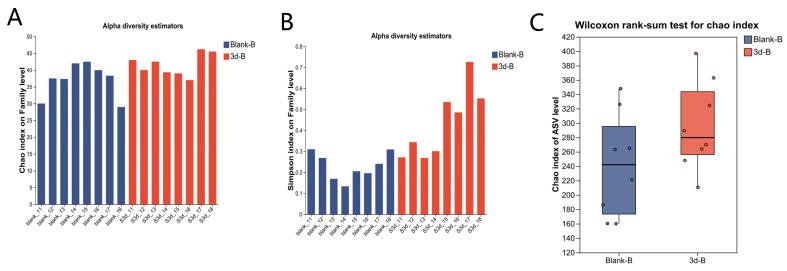

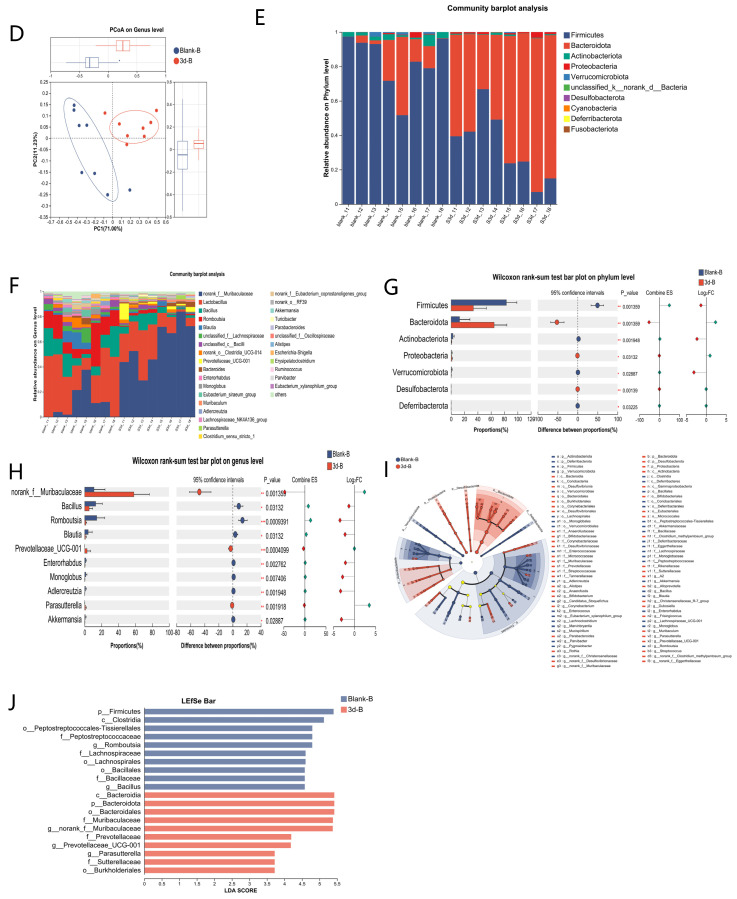
Effects of ionizing radiation (IR) on gut microbiota composition and diversity. (**A**) Chao index. (**B**) Shannon index. (**C**) Chao index test. (**D**) PCoA analysis on Genus level. (**E**) Relative abundances of the gut microbiota at the phylum level. (**F**) Relative abundances of the gut microbiota at the genus level. (**G**) Species difference analysis at the phylum level. (**H**) Species difference analysis at the genus level. (**I**) LEfSe multi-level species hierarchical tree diagram, using different colors to represent certain enriched taxa. (**J**) Linear discriminant analysis column chart. Only taxa with an LDA threshold value > 3 and showing significance are shown. * 0.01 < *p* ≤ 0.05, ** 0.001 < *p* ≤ 0.01, *** *p* ≤ 0.001.

Gut microbiota changes post-IR were assessed by 16S rRNA sequencing in control (Blank-B) and 3-day irradiated (3 d-B) fecal samples. The effects of ionizing radiation on intestinal bacterial diversity in rats were further analyzed using *α-* and *β*-diversity analyses. For α-diversity, the Chao (richness) and Shannon (diversity) indices are presented in Figure 5A–C. The results showed no significant changes in these indices. Rarefaction curves of ASV-level *α*-diversity indices—species richness (Sobs) and diversity (Simpson index)—demonstrated that both metrics plateaued with increasing sequencing depth across all groups (Figure S5), confirming data robustness. Principal coordinate analysis (PCoA) was performed to evaluate the β-diversity of the gut microbiota, showing a clear separation between the Blank-B and 3 d-B groups (Figure 5D). At the phylum level, two dominant taxa were identified: *Firmicutes* and *Bacteroidota* (Figure 5E). Differential abundance analysis revealed a significant increase in *Bacteroidota* abundance and a concurrent decrease in *Firmicutes* abundance post-irradiation (Figure 5F). Genus-level profiling identified the top 10 most abundant bacterial taxa: *norank_f_Muribaculaceae*, *Lactobacillus*, *Bacillus*, *Romboutsia*, *Blautia, unclassified_f_Lachnospiraceae*, *unclassified_c_Bacilli, norank_o_Clostridia_UCG_014*, *Prevotellaceae_UCG-001*, and *Bacteroides* (Figure 5G). The results of the differential analysis showed that the abundance of norank_f_Muribaculaceae increased significantly after irradiation, while the abundance of Bacillus and Romboutsia decreased significantly (Figure 5H). To investigate the distinctive microbial signatures between the Blank-B and 3 d-B groups, we conducted a three-dimensional Linear Discriminant Analysis (LDA) Effect Size (LEfSe) analysis with LDA scores >3. The Blank-B group exhibited characteristic *Firmicutes*-dominated microbiota (phylum), particularly represented by *Clostridia* (class) and *Peptostreptococcales-Tissierellales* (order). In contrast, the 3 d-B group demonstrated *Bacteroidia* predominance (class), with *Bacteroidota* (phylum) and Bacteroidales (order) as significantly enriched taxa (Figure 5I,J).

### 3.5. Combined Analysis of Microbiome and Metabolomics

**Figure 6:**
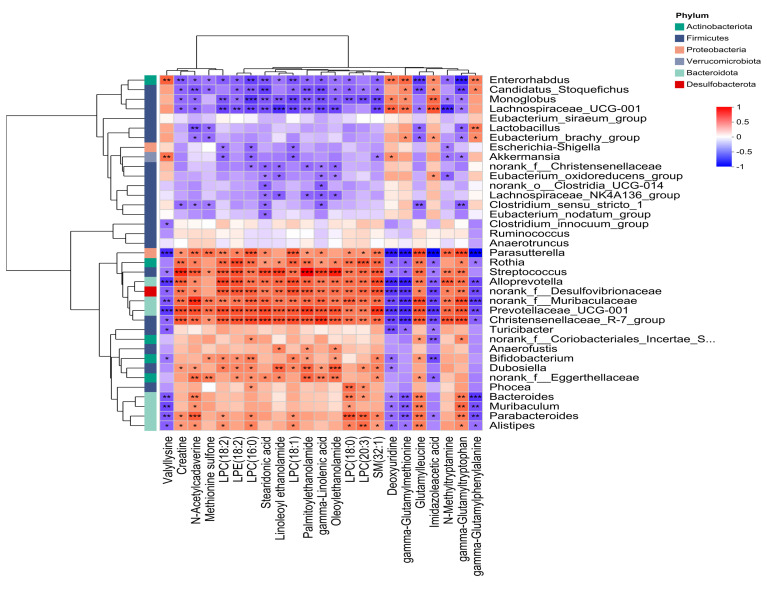
Correlation analysis between differential metabolites and differential gut microbiota. * 0.01 < *p* ≤ 0.05, ** 0.001 < *p* ≤ 0.01, *** *p* ≤ 0.001. Red hues denote statistically positive correlations; blue hues represent negative correlations.

To investigate metabolite–microbiota associations, we performed Spearman’s rank correlation analysis between the top 20 fecal metabolites (ranked by VIP scores and fold-change) and the top 50 most abundant bacterial taxa. Significant correlations were observed between radiation-altered metabolites and shifts in gut microbiota composition (Figure 6). *Norank_f_Muribaculaceae* negatively correlated with gamma-glutamylphenylalanine and gamma-glutamylmethionine (*p* < 0.001), while positively correlating with N-acetylcadaverine, LPC(16:0), LPC(18:0), glutamylleucine, and gamma-glutamytryptophan (*p* <0.001). *Prevotellaceae_UCG-001* showed negative associations with valyllysine, deoxyuridine, gamma-glutamylmethionine, imidazoleacetic acid, and gamma-glutamylphenylalanine (*p* < 0.01), but positive correlations with creatine, N-acetylcadaverine, LPC(18:2), LPE(18:2), LPC(16:0), stearidonic acid, linoleoyl ethanolamide, LPC(18:1), palmitoylethanolamide, gamma-Linolenic acid, oleoylethanolamide, SM(32:1), glutamylleucine, and gamma-glutamyltryptophan (*p* < 0.001). *Bacteroides* exhibited negative correlations with gamma-glutamylphenylalanine (*p* <0.001) and positive associations with N-acetylcadaverine, LPC (18:0), glutamylleucine, and gamma-glutamyltryptophan (*p* < 0.01).

## 4. Discussion

This study examined the temporal dynamics of metabolic alterations in Sprague-Dawley (SD) rats exposed to total-body γ-irradiation (7 Gy). The results demonstrated radiation-induced, time-dependent perturbations in hematopoietic indices, along with coordinated shifts in plasma and fecal metabolic profiles, identifying key metabolic pathways and microbiota taxa linked to radiation stress responses and recovery processes. Concurrently, gut microbiota profiling at 3 days post-irradiation revealed substantial taxonomic composition changes, including altered microbial diversity and abundance patterns. These findings advance our understanding of the systemic relationship between post-radiation metabolic reprogramming and gut dysbiosis, corroborating and extending the existing literature on radiation-associated biological cascades.

Radiation-induced hematopoietic suppression, evidenced by the nadir of blood cell counts at 14 days and subsequent recovery by 28 days, aligns with established models of acute radiation syndrome [30,31]. The transient weight loss observed during the first 14 days further underscores the systemic impact of radiation on energy homeostasis, consistent with reports linking ionizing radiation to catabolic states and metabolic stress [32,33]. Notably, the partial restoration of hematopoietic function and body weight by 28 days suggests activation of endogenous repair mechanisms, potentially mediated by redox-sensitive pathways such as glutathione metabolism [34], which was perturbed in our metabolomics data.

Metabolomic analysis revealed spatiotemporally distinct alterations in plasma and fecal samples following IR treatment, providing critical insights into systemic versus localized metabolic responses. Analysis of the above differential metabolites showed that after radiation, the differential metabolites were concentrated in the pathways of lipid metabolism disorder and cell membrane damage. Multiple subtypes of phosphatidylethanolamine (PE) and lysophospholipids (LPE/LPC), such as PE(38:5), LPE(18:2), and LPE(14:0), showed significant changes. This observation aligns with existing research findings that PE, LPE, and LPC undergo substantial alterations following radiation exposure [35,36]. The results suggest that radiation might disrupt the cell membrane structure through lipid peroxidation [37]. PE is a major component of the cell membrane. Its hydrolysis to generate lysophospholipids may activate inflammatory signaling pathways (such as NF-κB), exacerbating the oxidative stress response. Altered levels of sphingomyelin (SM(34:0)) and ceramide phosphate (CerP(30:1)) were observed, consistent with prior research documenting radiation-induced perturbations in sphingolipid profiles [35,36]. These changes suggest radiation-induced reprogramming of sphingolipid metabolism [38]. As a long-chain fatty acid, nervonic acid is involved in myelin formation, and its decrease may imply damage to nerve cells [39]. There were also many differential metabolites in the pathways of mitochondrial dysfunction and energy metabolism. Significant changes in short-chain and medium-chain acylcarnitines (such as methylmalonylcarnitine and glutarylcarnitine), consistent with previous reports of radiation-induced perturbations in lipid metabolism [9,10,11], suggested that mitochondrial β-oxidation was blocked, possibly due to radiation damage to the mitochondrial membrane. The increase in methylmalonylcarnitine may be associated with abnormal Vitamin B12 metabolism, leading to a disorder in succinyl-CoA synthesis and further affecting the tricarboxylic acid (TCA) cycle [40]. Spermidine, belonging to polyamine substances, has antioxidant and autophagy-regulating functions. Its changes may reflect that cells remove radiation-induced damaged proteins or organelles by activating the autophagy pathway [41,42]. Many differential metabolites were found in oxidative stress and inflammatory responses. The decrease in unsaturated fatty acids, such as palmitoleic acid (ω-7 fatty acid), may be related to the depletion of the antioxidant defense system. The tryptophan derivative tryptophan-2-C-mannoside may participate in immune regulation through the kynurenine pathway—a metabolic axis increasingly recognized for its roles in radiation response [20]—and its changes may be associated with radiation-induced immunosuppression or the release of inflammatory factors (such as IL-6 and TNF-α) [43]. The core pathways of the above differential metabolites include fatty acid β-oxidation (abnormal carnitine metabolism), sphingolipid signaling pathway (apoptosis mediated by CerP/SM), phospholipid remodeling (imbalance of PE/LysoPE), and the oxidative stress–autophagy axis (spermidine and palmitoleic acid).

Through the analysis of the differential metabolites in feces after radiation, the top 20 metabolites in terms of fold change and VIP (Variable Importance in the Projection) values are mainly involved in processes such as energy metabolism, oxidative stress, membrane phospholipid remodeling, inflammatory regulation, and DNA repair. Among them, the significant changes in creatine may reflect the interference of radiation on muscle energy metabolism. Creatine is responsible for the regeneration of ATP in the phosphocreatine–creatine kinase system. Radiation may lead to an imbalance in creatine/creatinine metabolism by inhibiting mitochondrial function or inducing muscle atrophy [44]. Methionine sulfone is an oxidation product of methionine, and its accumulation directly indicates that radiation-induced reactive oxygen species (ROS) attack sulfur-containing amino acids [45]. The oxidation of methionine may weaken its function as a methyl donor, affecting epigenetic regulation and protein synthesis. Gamma-Glutamylmethionine, as an intermediate in glutathione metabolism, further supports the activation of the oxidative stress defense system [46]. Linoleoyl ethanolamide and palmitoylethanolamide belong to fatty acid ethanolamides, which may inhibit the inflammatory response by activating peroxisome proliferator-activated receptor (PPAR-α) or endogenous cannabinoid receptors [47]. Meanwhile, there are changes in substances related to nucleotide metabolism and DNA repair after radiation. The enrichment of ethanolamides such as oeoylethanolamide indicates that the endogenous cannabinoid system may be involved in metabolic adaptation after radiation. These molecules can regulate appetite, energy metabolism, and inflammation by binding to G protein-coupled receptors (such as GPR119) [48], but their specific roles in radiation protection still need in-depth exploration.

Three days after the irradiation treatment, *Firmicutes* and *Bacteroidota* have always been in a dominant position, and they show a significant reverse change trend: irradiation leads to a continuous increase in the abundance of *Bacteroidota* (*p* < 0.01), whereas the abundance of *Firmicutes* decreases significantly (*p* < 0.01)—a reciprocal shift consistent with radiation-associated dysbiosis patterns reported in prior animal studies [21,22]. This phenomenon suggests that irradiation may selectively inhibit the proliferation of *Firmicutes* by altering the host metabolic environment (such as the levels of short-chain fatty acids) or the intestinal pH value, while simultaneously promoting the colonization of Bacteroidota. The decrease in the *Firmicutes/Bacteroidota* (F/B) ratio may be associated with the regulation of intestinal inflammation or energy metabolism [49]. Three days after radiation, the abundances of the family *Muribaculaceae* (*norank_f_Muribaculaceae*) (*p* < 0.001) and the genus *Prevotellaceae_UCG-001* (*p* < 0.05) increased significantly. They may regulate lipid metabolism, produce short-chain fatty acids (SCFAs), and reduce inflammation [50,51]. Bacillus may maintain long-term survival through sporulation, but reactivation of its metabolic activity post-radiation is challenging in the short term [52]. This impairment compromises key host health-related functions. Specifically, *Bacillus* ferments dietary fibers to produce short-chain fatty acids (SCFAs), such as acetate, propionate, and butyrate, which are essential for gut homeostasis and energy metabolism [53]. Radiation exposure disrupts this process, leading to reduced SCFA synthesis. Additionally, *Romboutsia*—a genus critical for colonic butyrate production through enzymatic conversion of dietary fiber—plays a pivotal role in SCFA generation. The biochemical interplay between Romboutsia and SCFA production may be perturbed by radiation-induced dysregulation of energy metabolism, which could further inhibit its functional capacity [54]. Therefore, the abundances of the above-mentioned genera decrease after radiation (*p* < 0.05, *p* < 0.001). The reduction in lactic acid-producing bacteria, notably the genus *Lactobacillus* (*p* < 0.05), may impair intestinal acidification. This decline in lactic acid synthesis likely destabilizes the gut’s pH equilibrium, resulting in an elevated luminal pH. Such alkaline conditions promote the proliferation of pathogenic and opportunistic bacteria, which preferentially colonize less acidic environments [55].

The present study revealed significant correlations between differential fecal metabolites and microbiota, suggesting potential functional interactions between gut microbial communities and host metabolic pathways. The observed negative correlations between *Norank_f_Muribaculaceae*/*Prevotellaceae_UCG-001* and gamma-glutamyl amino acids (e.g., gamma-glutamylphenylalanine/methionine) may reflect microbial modulation of host glutathione metabolism. Gamma-glutamyltransferase (GGT)-mediated cleavage of glutathione generates these metabolites, which are elevated under oxidative stress [56]. The suppression of gamma-glutamyl derivatives by specific taxa suggests a potential antioxidant role, possibly via microbial regulation of GGT activity or glutathione homeostasis. Conversely, the positive associations of *Bacteroides* and *Prevotellaceae_UCG-001* with lysophosphatidylcholines (LPCs) and endocannabinoids (e.g., palmitoylethanolamide) highlight microbiota-driven lipid signaling pathways. LPCs serve as precursors for pro-inflammatory mediators, while endocannabinoids modulate immune responses via PPAR-α activation [57]. These interactions may underpin gut barrier integrity and systemic inflammation regulation. The robust positive associations of N-Acetylcadaverine with diverse microbial taxa highlight its significance as a polyamine derivative linked to gut dysbiosis-related pathologies. Its microbial biosynthesis (e.g., Bacteroides-driven lysine decarboxylation) positions it as a potential diagnostic biomarker for disorders associated with disrupted gut microbiota homeostasis [58]. Similarly, the co-enrichment of sphingomyelin SM(32:1) and endocannabinoids with *Prevotellaceae_UCG-001* suggests a role in membrane signaling and TLR pathway modulation, critical for mucosal immunity [59]. Targeting these metabolites via probiotics or dietary interventions (e.g., omega-3 supplementation to boost stearidonic acid) may offer therapeutic strategies for metabolic disorders.

The convergence of sphingolipid and arachidonic acid metabolism pathways in plasma and feces highlights systemic inflammatory responses. Sphingolipids regulate apoptosis and immune cell trafficking, while arachidonic acid derivatives (e.g., prostaglandins) are central to radiation-induced pro-inflammatory signaling [60]. Notably, the temporal divergence in plasma (peak at 14 days) versus fecal (peak at 3 days) metabolic disruptions suggests compartment-specific recovery trajectories, possibly influenced by differential microbial resilience [61]. This observation underscores the need for multi-omics longitudinal studies to unravel organ-specific repair mechanisms.

Despite these insights, limitations must be acknowledged. The small sample size (n = 8 per group) may limit statistical power, particularly in detecting subtle microbiota changes. Future studies with a larger number of animals per group are warranted to improve statistical power and better capture such subtle changes. A further limitation is the exclusive use of male rats, which may restrict the generalizability of sex-sensitive metabolites. For instance, xanthurenic acid levels are known to fluctuate with estrogen cycles in females [62], suggesting potential sex-specific responses to radiation. While this design minimizes hormonal variability, future work must include female cohorts to validate whether the observed metabolic and microbiome shifts are sex-dependent. Structural confirmation of metabolites requires direct comparison with authentic standards. Additionally, while metabolomics identified pathway-level perturbations, mechanistic validation (e.g., knockouts or isotopic tracing) is required to establish causality. Future studies should integrate transcriptomic or proteomic data to elucidate regulatory networks linking metabolites, microbiota, and radiation responses. While MS/MS spectral matching provided confident annotations, a key limitation of this study is the reliance on public database annotations for metabolite identification, as experimental validation using chemical standards was not performed. Future studies will focus on structural confirmation using authentic chemical standards to validate metabolite identities, followed by targeted metabolomics approaches to rigorously quantify dynamic changes in critical metabolites. This dual strategy will enhance the reliability of biomarker discovery and mechanistic insights.

## 5. Conclusions

In conclusion, our study delineates critical metabolic and microbial signatures of radiation injury and recovery, emphasizing the interplay between host metabolism and gut microbiota. These findings advance the understanding of ARS pathophysiology and may inform therapeutic strategies targeting metabolic pathways or microbial taxa to mitigate radiation toxicity.

## Figures and Tables

**Table 1:** KEGG pathway enrichment analysis of plasma metabolome perturbations.

Pathway Name	−log(*p*)	Impact
Glycerophospholipid metabolism	2.7719	0.21708
Alpha-linolenic acid metabolism	2.4622	0.33333
Biosynthesis of unsaturated fatty acids	1.5938	0
Arginine and proline metabolism	1.5938	0.06628
Linoleic acid metabolism	1.4539	0
Arginine biosynthesis	1.0194	0
Beta-alanine metabolism	0.85305	0
Glutathione metabolism	0.73783	0.00719
Lysine degradation	0.71063	0
Sphingolipid metabolism	0.68536	0
Glycosylphosphatidylinositol (GPI)-anchor biosynthesis	0.68536	0.03665
Pyrimidine metabolism	0.60909	0.0079
Arachidonic acid metabolism	0.57217	0

**Table 2:** KEGG pathway enrichment analysis of feces metabolome perturbations.

Pathway Name	−log(*p*)	Impact
Tryptophan metabolism	3.4321	0.25291
Phenylalanine, tyrosine, and tryptophan biosynthesis	2.8294	0.5
Biosynthesis of unsaturated fatty acids	2.653	0
Linoleic acid metabolism	2.6119	1
Vitamin B6 metabolism	2.0729	0.15686
Alpha-linolenic acid metabolism	1.7543	0
Tyrosine metabolism	1.5468	0.24711
Ubiquinone and other terpenoid-quinone biosynthesis	1.4392	0.04545
Glycerophospholipid metabolism	0.94507	0.11201
Valine, leucine, and isoleucine biosynthesis	0.9112	0
Phenylalanine metabolism	0.82096	0
Histidine metabolism	0.63666	0
Purine metabolism	0.49398	0.01316
One carbon pool by folate	0.45824	0.05077
Sphingolipid metabolism	0.38715	0.21576
Cysteine and methionine metabolism	0.37694	0.10446
Glycine, serine, and threonine metabolism	0.36712	0
Arginine and proline metabolism	0.34857	0.02442
Pyrimidine metabolism	0.32315	0.03799
Valine, leucine, and isoleucine degradation	0.31526	0
Arachidonic acid metabolism	0.2931	0

## Data Availability

The original contributions presented in this study are included in the article and Supplementary Materials. Further inquiries can be directed to the corresponding author.

## Supplementary Materials

The following supporting information can be downloaded at: https://www.mdpi.com/article/10.3390/metabo15060363/s1, Figure S1: Temporal changes of the top 20 substances with the highest fold-change ratios in plasma; Figure S2: Temporal changes of the top 20 compounds ranked by VIP values in the radiation group in plasma; Figure S3: Temporal changes of the top 20 compounds ranked by fold change in feces; Figure S4: Temporal changes of the top 20 compounds ranked by VIP values in the radiation group in feces; Figure S5: Rarefaction curves.
