# Supplementary material for: Plasma and Fecal Metabolites Combined with Gut Microbiome Reveal Systemic Metabolic Shifts in 60Co Gamma-Irradiated Rats
Source: Metabolites. 2025 May 29;15(6):363. doi: 10.3390/metabo15060363 (PMC12194991; doi:10.3390/metabo15060363)
Supplement: Supplementary file 1 [file metabolites-15-00363-s001.zip › Supplementary Figures.pdf]

(a)

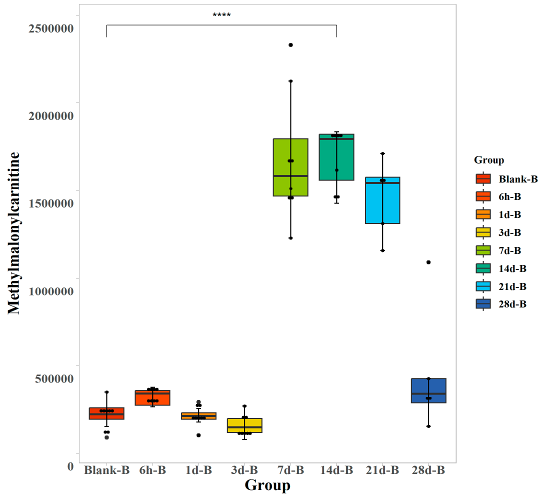
****
Methylmalonylcarnitine
2500000
2000000
1500000
1000000
500000
0
Blank-B 6h-B 1d-B 3d-B 7d-B 14d-B 21d-B 28d-B
Group
Group
Blank-B
6h-B
1d-B
3d-B
7d-B
14d-B
21d-B
28d-B

(b)

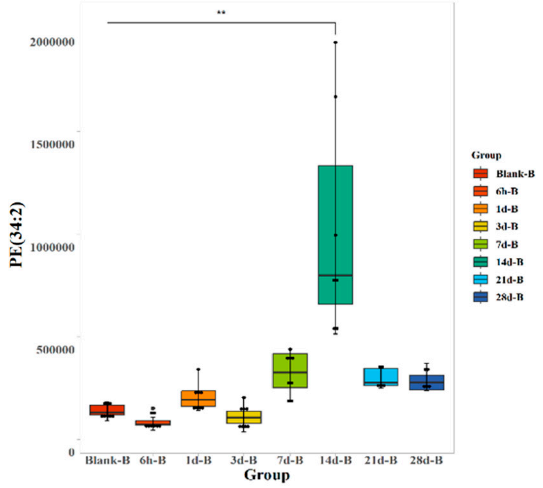
**
PE(34:2)
2000000
1500000
1000000
500000
0
Blank-B 6h-B 1d-B 3d-B 7d-B 14d-B 21d-B 28d-B
Group
Group
Blank-B
6h-B
1d-B
3d-B
7d-B
14d-B
21d-B
28d-B

(c)

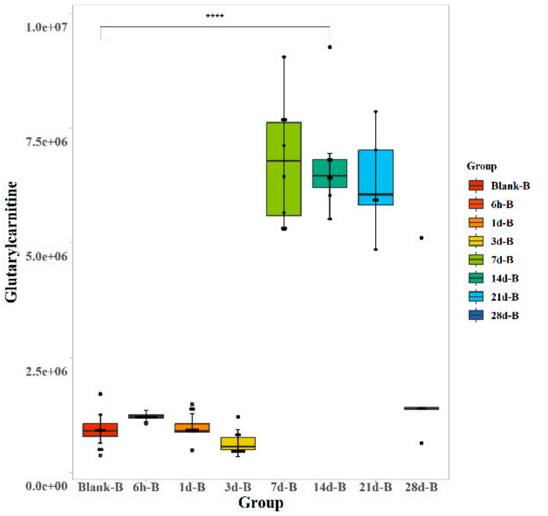
****
Glutarylcarnitine
1.0e+07
7.5e+06
5.0e+06
2.5e+06
0.0e+00
Blank-B 6h-B 1d-B 3d-B 7d-B 14d-B 21d-B 28d-B
Group
Group
Blank-B
6h-B
1d-B
3d-B
7d-B
14d-B
21d-B
28d-B

(d)

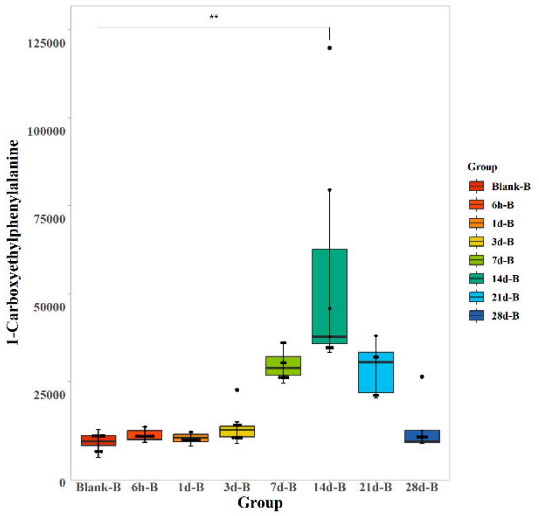
**
1-Carboxyethylphenylalanine
125000
100000
75000
50000
25000
0
Blank-B 6h-B 1d-B 3d-B 7d-B 14d-B 21d-B 28d-B
Group
Group
Blank-B
6h-B
1d-B
3d-B
7d-B
14d-B
21d-B
28d-B

(e)

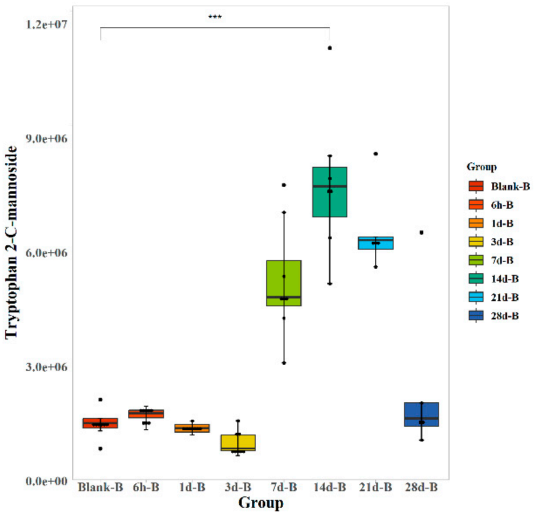
***
Tryptophan 2-C-mannoside
1.2e+07
9.0e+06
6.0e+06
3.0e+06
0.0e+00
Blank-B 6h-B 1d-B 3d-B 7d-B 14d-B 21d-B 28d-B
Group
Group
Blank-B
6h-B
1d-B
3d-B
7d-B
14d-B
21d-B
28d-B

(f)

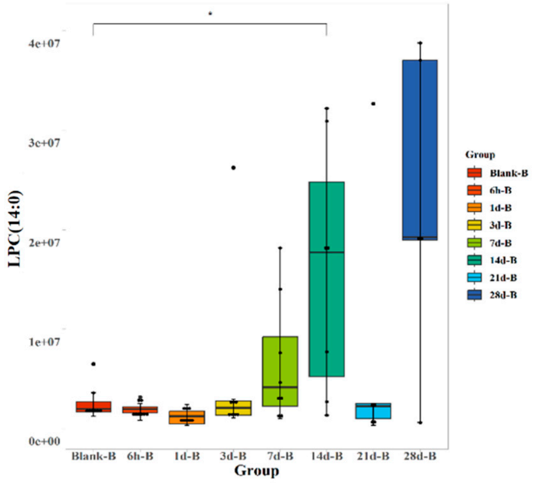
*
LPC(14:0)
4e+07
3e+07
2e+07
1e+07
0e+00
Blank-B 6h-B 1d-B 3d-B 7d-B 14d-B 21d-B 28d-B
Group
Group
Blank-B
6h-B
1d-B
3d-B
7d-B
14d-B
21d-B
28d-B

(g)

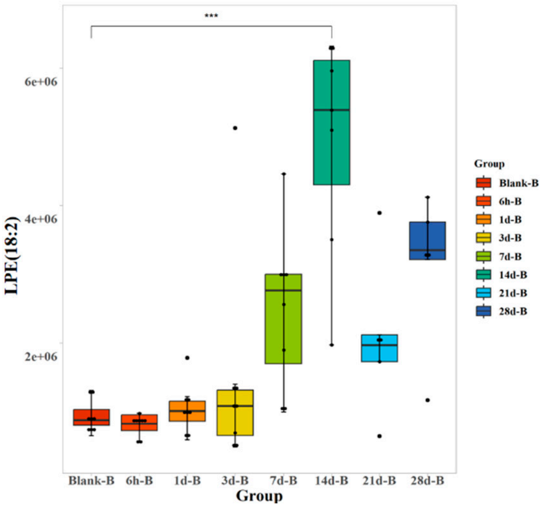

***
LPE(18:2)
6e+06
4e+06
2e+06
Blank-B 6h-B 1d-B 3d-B 7d-B 14d-B 21d-B 28d-B
Group
Group
Blank-B
6h-B
1d-B
3d-B
7d-B
14d-B
21d-B
28d-B

(h)

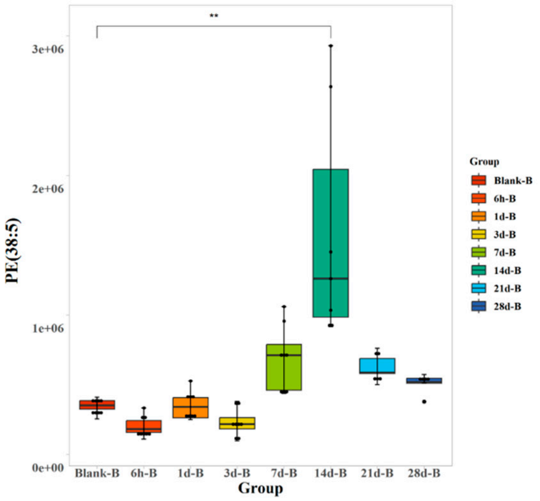

**
PE(38:5)
3e+06
2e+06
1e+06
0e+00
Blank-B 6h-B 1d-B 3d-B 7d-B 14d-B 21d-B 28d-B
Group
Group
Blank-B
6h-B
1d-B
3d-B
7d-B
14d-B
21d-B
28d-B

(i)

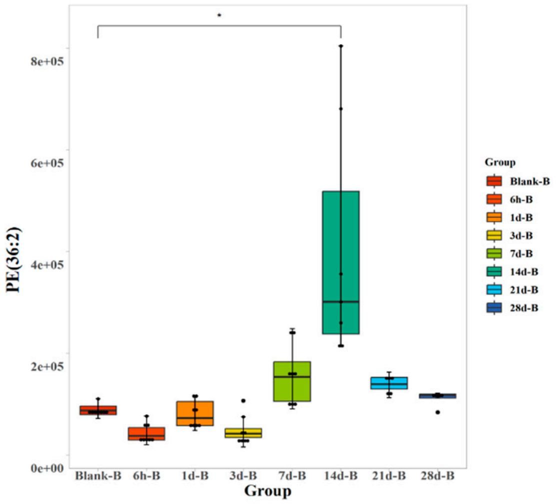

*
PE(36:2)
8e+05
6e+05
4e+05
2e+05
0e+00
Blank-B 6h-B 1d-B 3d-B 7d-B 14d-B 21d-B 28d-B
Group
Group
Blank-B
6h-B
1d-B
3d-B
7d-B
14d-B
21d-B
28d-B

(j)

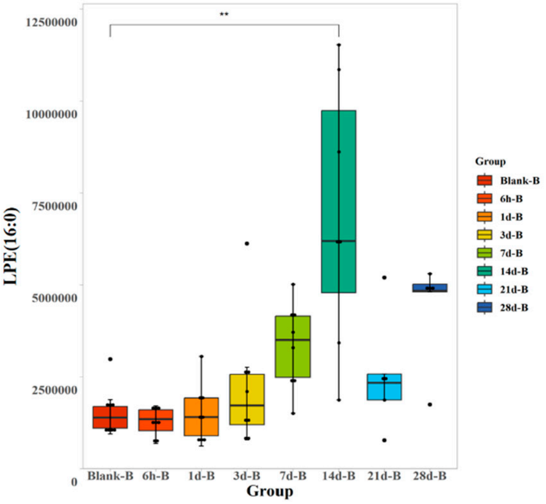

**
LPE(16:0)
12500000
10000000
7500000
5000000
2500000
0
Blank-B 6h-B 1d-B 3d-B 7d-B 14d-B 21d-B 28d-B
Group
Group
Blank-B
6h-B
1d-B
3d-B
7d-B
14d-B
21d-B
28d-B

(k)

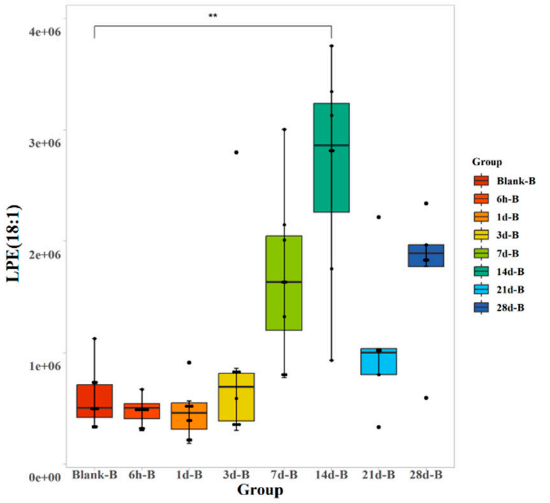

**
LPE(18:1)
4e+06
3e+06
2e+06
1e+06
0e+00
Blank-B 6h-B 1d-B 3d-B 7d-B 14d-B 21d-B 28d-B
Group
Group
Blank-B
6h-B
1d-B
3d-B
7d-B
14d-B
21d-B
28d-B

(l)

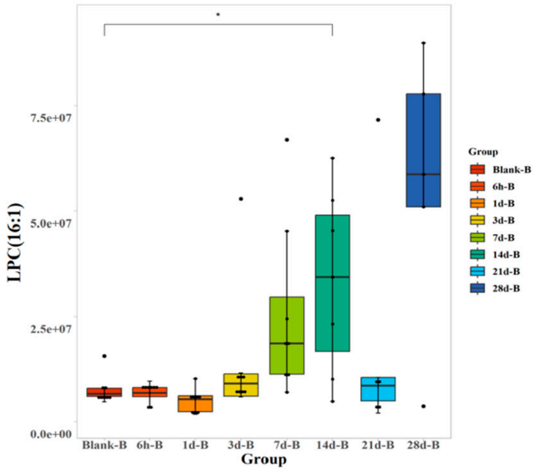

*
LPC(16:1)
7.5e+07
5.0e+07
2.5e+07
0.0e+00
Blank-B 6h-B 1d-B 3d-B 7d-B 14d-B 21d-B 28d-B
Group
Group
Blank-B
6h-B
1d-B
3d-B
7d-B
14d-B
21d-B
28d-B

(m)

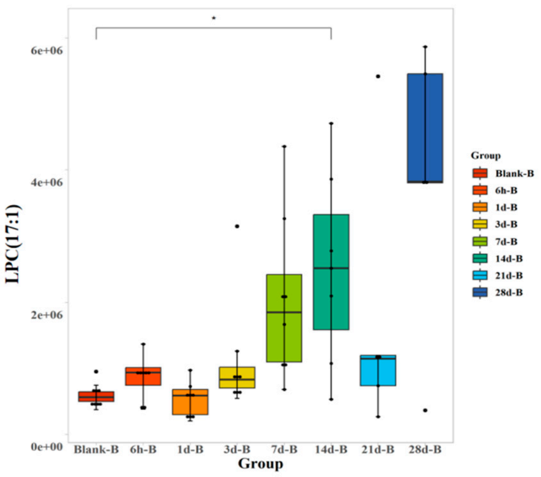

*
6e+06
4e+06
2e+06
0e+00
LPC(17:1)
Blank-B 6h-B 1d-B 3d-B 7d-B 14d-B 21d-B 28d-B
Group
Group
Blank-B
6h-B
1d-B
3d-B
7d-B
14d-B
21d-B
28d-B

(n)

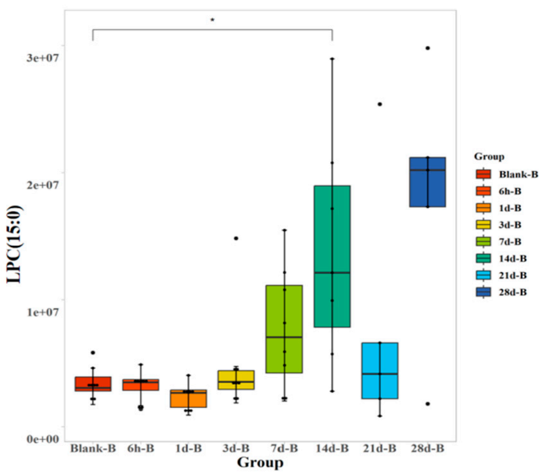

*
3e+07
2e+07
1e+07
0e+00
LPC(15:0)
Blank-B 6h-B 1d-B 3d-B 7d-B 14d-B 21d-B 28d-B
Group
Group
Blank-B
6h-B
1d-B
3d-B
7d-B
14d-B
21d-B
28d-B

(o)

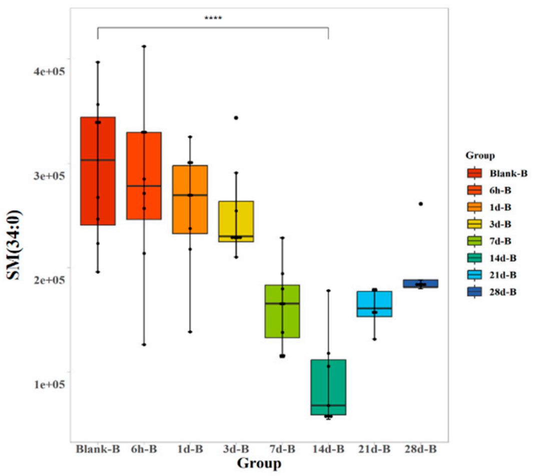

****
4e+05
3e+05
2e+05
1e+05
SM(34:0)
Blank-B 6h-B 1d-B 3d-B 7d-B 14d-B 21d-B 28d-B
Group
Group
Blank-B
6h-B
1d-B
3d-B
7d-B
14d-B
21d-B
28d-B

(p)

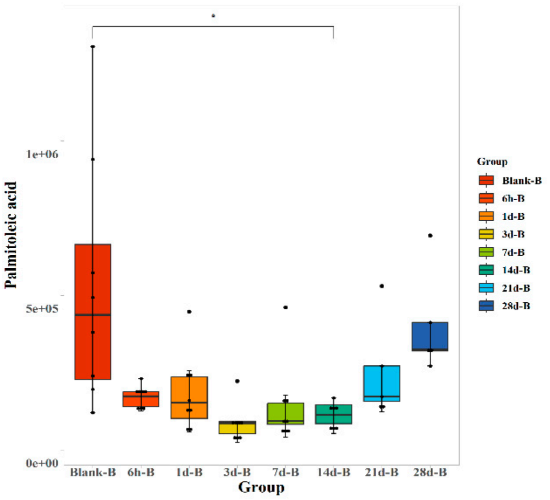

*
1e+06
5e+05
0e+00
Palmitoleic acid
Blank-B 6h-B 1d-B 3d-B 7d-B 14d-B 21d-B 28d-B
Group
Group
Blank-B
6h-B
1d-B
3d-B
7d-B
14d-B
21d-B
28d-B

(q)

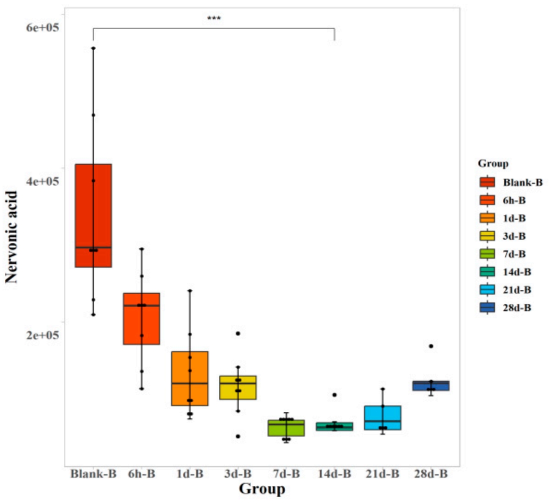

***
6e+05
4e+05
2e+05
Nervonic acid
Blank-B 6h-B 1d-B 3d-B 7d-B 14d-B 21d-B 28d-B
Group
Group
Blank-B
6h-B
1d-B
3d-B
7d-B
14d-B
21d-B
28d-B

(r)

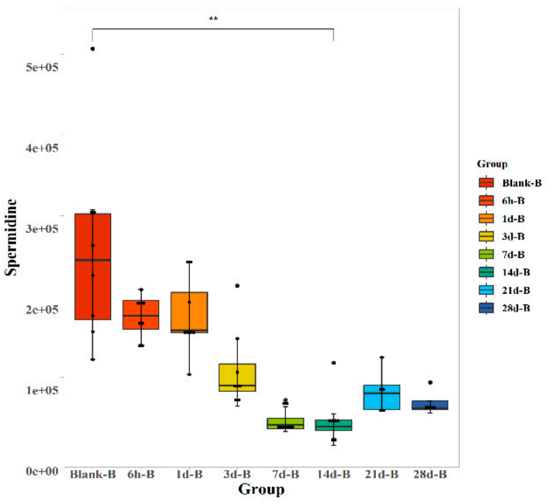

**
5e+05
4e+05
3e+05
2e+05
1e+05
0e+00
Spermidine
Blank-B 6h-B 1d-B 3d-B 7d-B 14d-B 21d-B 28d-B
Group
Group
Blank-B
6h-B
1d-B
3d-B
7d-B
14d-B
21d-B
28d-B

(s)

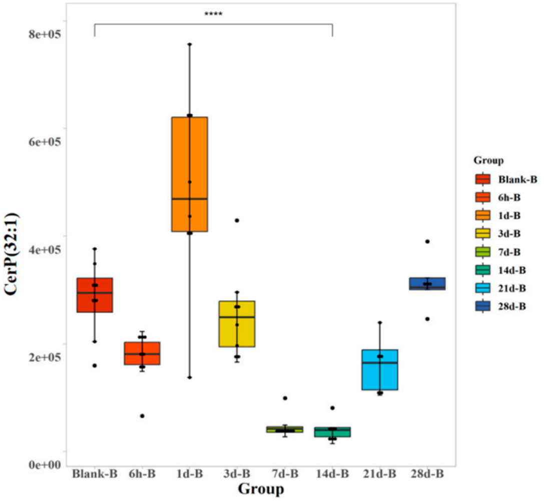

(t)

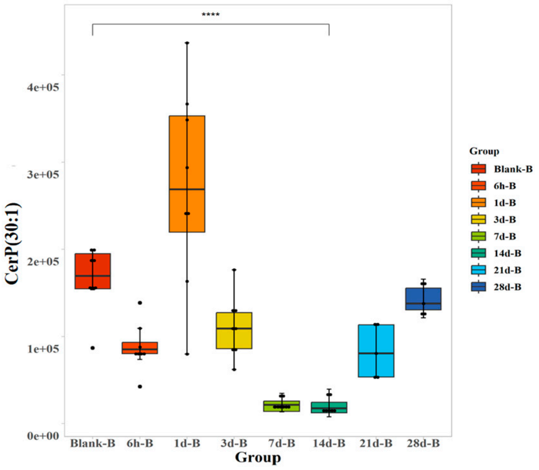

Supplementary Figure S1. Temporal changes of the top 20 substances with the highest fold-change ratios in plasma

(a)Methylmalonylcarnitine; (b)PE(34:2); (c)Glutarylcarnitine; (d)1-Carboxyethylbutoylalanine; (e)Tryptophan2-C-mannoside; (f)LPC(14:0); (g)LPE(18:2); (h)PE(38:5); (i)PE(36:2); (j)LPE(16:0); (k)LPE(18:1); (l)LPC(16:1); (m)LPC(17:1); (n)LPC(15:0); (o)SM(34:0); (p)Palmitoleic acid; (q)Nervonic acid; (r)Spermidine; (s)CerP(32:1); (t)CerP(30:1)

Radiation group (Group B); Blank-B (pre-radiation in Group B); 6h-B (6 hours post-radiation in Group B); 1d-B (1 day post-radiation in Group B); 3d-B (3 days post-radiation in Group B); 7d-B (7 days post-radiation in Group B); 14d-B (14 days post-radiation in Group B); 21d-B (21 days post-radiation in Group B); 28d-B (28 days post-radiation in Group B).

*Statistical significance vs. pre-radiation: *P < 0.05, **P < 0.01, ***P < 0.001.*

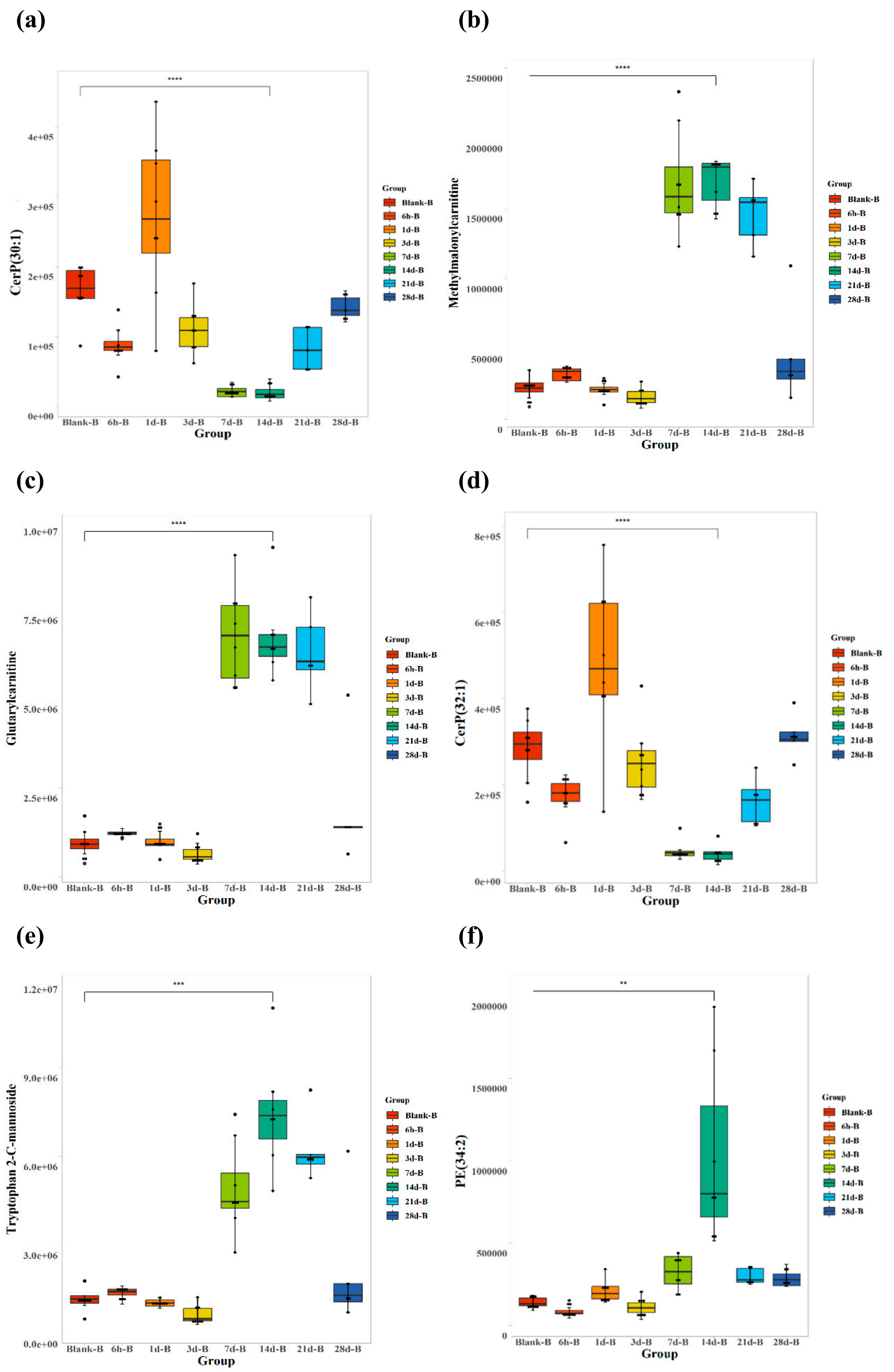

(a)
(b)
(c)
(d)
(e)
(f)
CerP(30:1)
Methylmalonylcarnitine
Glutarylcarnitine
CerP(32:1)
Tryptophan 2-C-mannoside
PE(34:2)
Group
Blank-B
6h-B
1d-B
3d-B
7d-B
14d-B
21d-B
28d-B
****
***
**

(g)

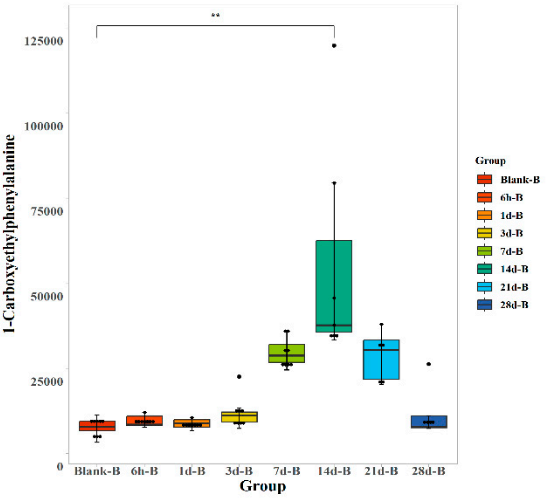
**
125000
100000
75000
50000
25000
0
1-Carboxyethylphenylalanine
Blank-B
6h-B
1d-B
3d-B
7d-B
14d-B
21d-B
28d-B
Group
Group
Blank-B
6h-B
1d-B
3d-B
7d-B
14d-B
21d-B
28d-B

(h)

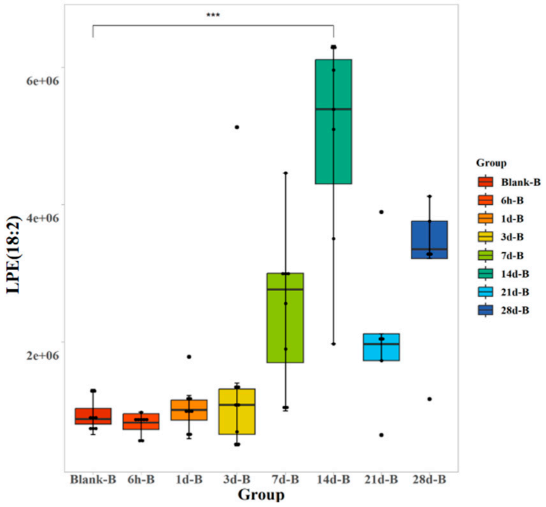
***
6e+06
4e+06
2e+06
LPE(18:2)
Blank-B
6h-B
1d-B
3d-B
7d-B
14d-B
21d-B
28d-B
Group
Group
Blank-B
6h-B
1d-B
3d-B
7d-B
14d-B
21d-B
28d-B

(i)

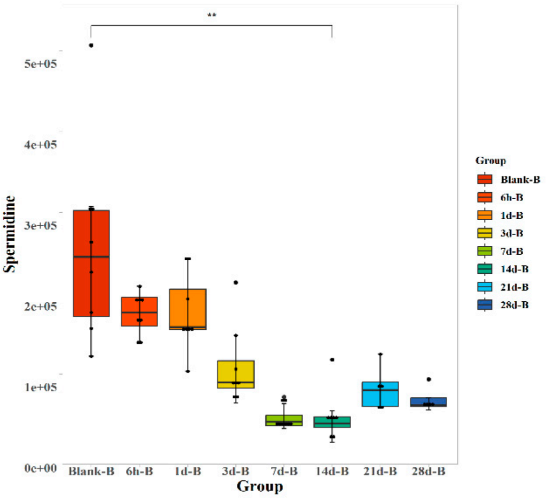
**
5e+05
4e+05
3e+05
2e+05
1e+05
0e+00
Spermidine
Blank-B
6h-B
1d-B
3d-B
7d-B
14d-B
21d-B
28d-B
Group
Group
Blank-B
6h-B
1d-B
3d-B
7d-B
14d-B
21d-B
28d-B

(j)

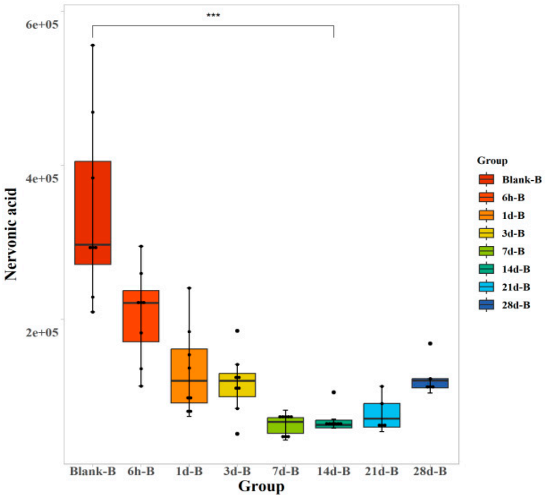
***
6e+05
4e+05
2e+05
Nervonic acid
Blank-B
6h-B
1d-B
3d-B
7d-B
14d-B
21d-B
28d-B
Group
Group
Blank-B
6h-B
1d-B
3d-B
7d-B
14d-B
21d-B
28d-B

(k)

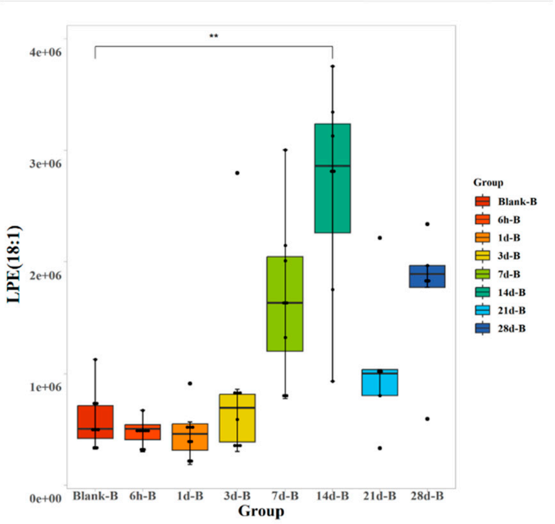
**
4e+06
3e+06
2e+06
1e+06
0e+00
LPE(18:1)
Blank-B
6h-B
1d-B
3d-B
7d-B
14d-B
21d-B
28d-B
Group
Group
Blank-B
6h-B
1d-B
3d-B
7d-B
14d-B
21d-B
28d-B

(l)

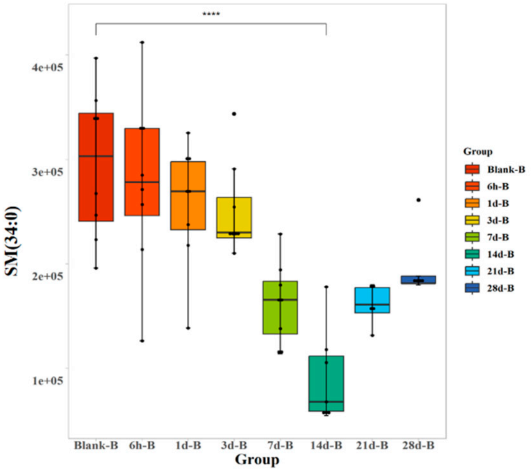
****
4e+05
3e+05
2e+05
1e+05
SM(34:0)
Blank-B
6h-B
1d-B
3d-B
7d-B
14d-B
21d-B
28d-B
Group
Group
Blank-B
6h-B
1d-B
3d-B
7d-B
14d-B
21d-B
28d-B

(m)

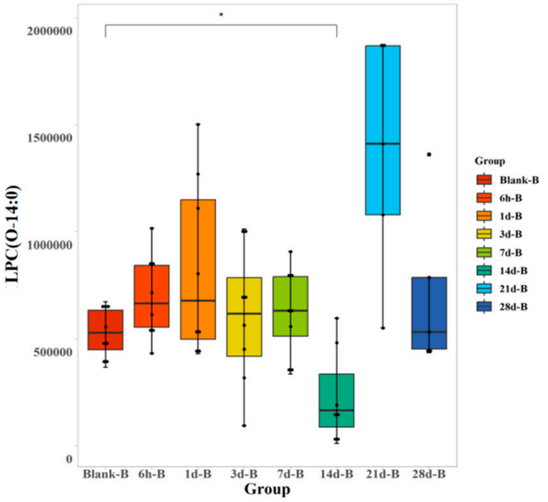

*
2000000
1500000
1000000
500000
0
LPC(O-14:0)
Blank-B 6h-B 1d-B 3d-B 7d-B 14d-B 21d-B 28d-B
Group
Group
Blank-B
6h-B
1d-B
3d-B
7d-B
14d-B
21d-B
28d-B

(n)

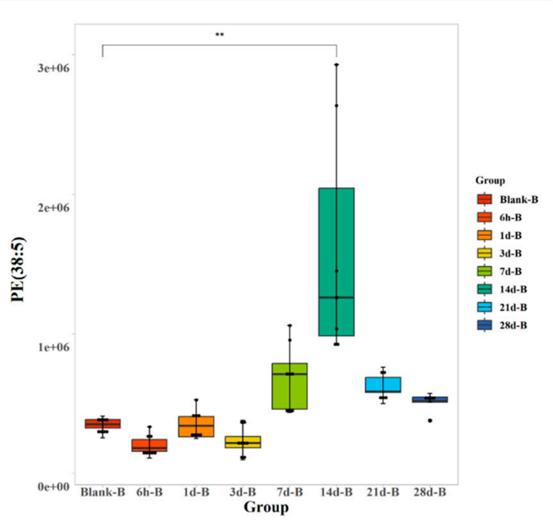

**
3e+06
2e+06
1e+06
0e+00
PE(38:5)
Blank-B 6h-B 1d-B 3d-B 7d-B 14d-B 21d-B 28d-B
Group
Group
Blank-B
6h-B
1d-B
3d-B
7d-B
14d-B
21d-B
28d-B

(o)

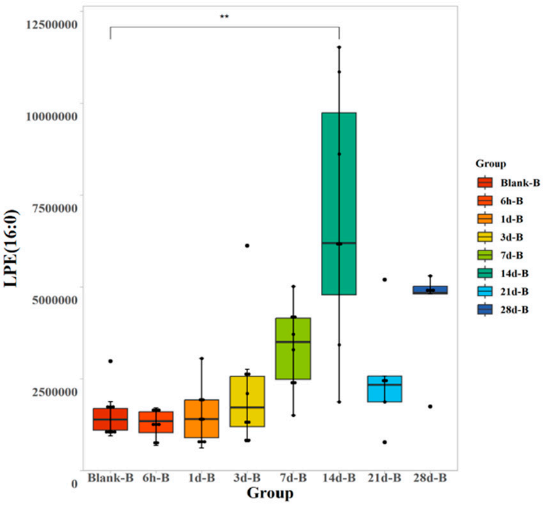

**
12500000
10000000
7500000
5000000
2500000
0
LPE(16:0)
Blank-B 6h-B 1d-B 3d-B 7d-B 14d-B 21d-B 28d-B
Group
Group
Blank-B
6h-B
1d-B
3d-B
7d-B
14d-B
21d-B
28d-B

(p)

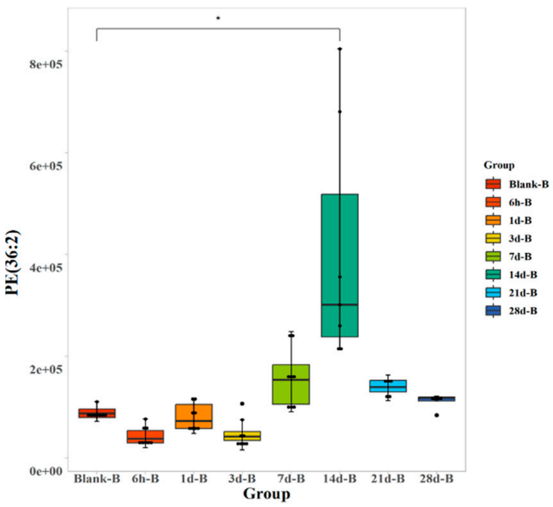

*
8e+05
6e+05
4e+05
2e+05
0e+00
PE(36:2)
Blank-B 6h-B 1d-B 3d-B 7d-B 14d-B 21d-B 28d-B
Group
Group
Blank-B
6h-B
1d-B
3d-B
7d-B
14d-B
21d-B
28d-B

(q)

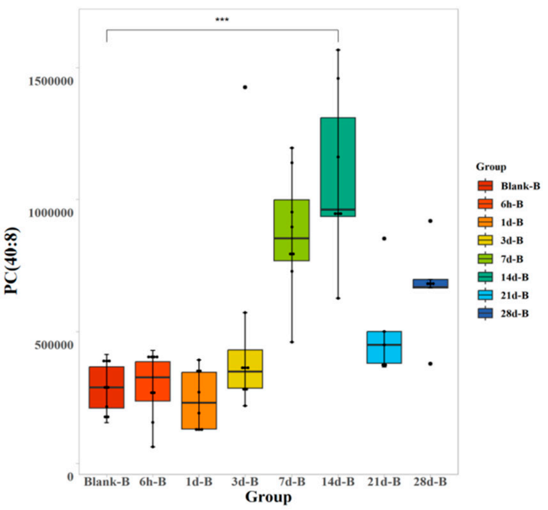

***
1500000
1000000
500000
0
PC(40:8)
Blank-B 6h-B 1d-B 3d-B 7d-B 14d-B 21d-B 28d-B
Group
Group
Blank-B
6h-B
1d-B
3d-B
7d-B
14d-B
21d-B
28d-B

(r)

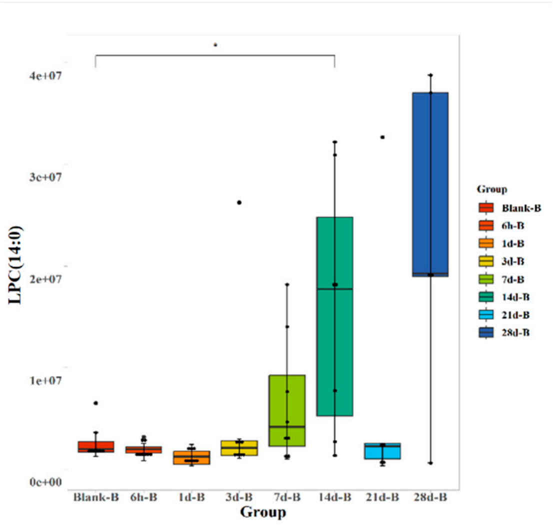

*
4e+07
3e+07
2e+07
1e+07
0e+00
LPC(14:0)
Blank-B 6h-B 1d-B 3d-B 7d-B 14d-B 21d-B 28d-B
Group
Group
Blank-B
6h-B
1d-B
3d-B
7d-B
14d-B
21d-B
28d-B

(s)

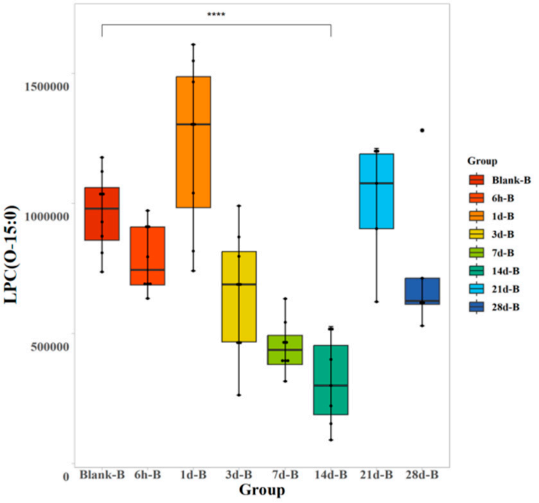

(t)

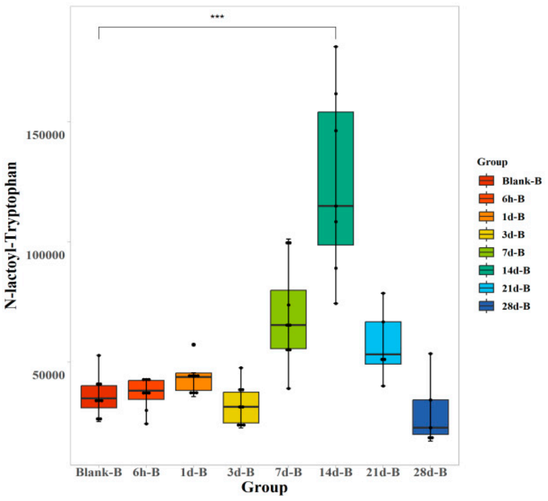

Supplementary Figure S2. Temporal changes of the top 20 compounds ranked by VIP values in the radiation group in plasma

(a)CerP(30:1); (b)Methylmalonylcarnitine; (c)Glutarylcarnitine; (d)CerP(32:1); (e)Tryptophan 2-C-mannoside; (f)PE(34:2); (g)1-Carboxyethylbutoylalanine; (h)LPE(18:2); (i)Spermidine; (j)Nervonicacid; (k)LPE(18:1); (l)SM(34:0); (m)LPC(O-14:0); (n)PE(38:5); (o)LPE(16:0); (p)PE(36:2); (q)PC(40:8); (r)LPC(14:0); (s)LPC(O-15:0); (t)N-lactoyl-Tryptophan

Radiation group (Group B); Blank-B (pre-radiation in Group B); 6h-B (6 hours post-radiation in Group B); 1d-B (1 day post-radiation in Group B); 3d-B (3 days post-radiation in Group B); 7d-B (7 days post-radiation in Group B); 14d-B (14 days post-radiation in Group B); 21d-B (21 days post-radiation in Group B); 28d-B (28 days post-radiation in Group B).

*Statistical significance vs. pre-radiation: *P < 0.05, **P < 0.01, ***P < 0.001.*

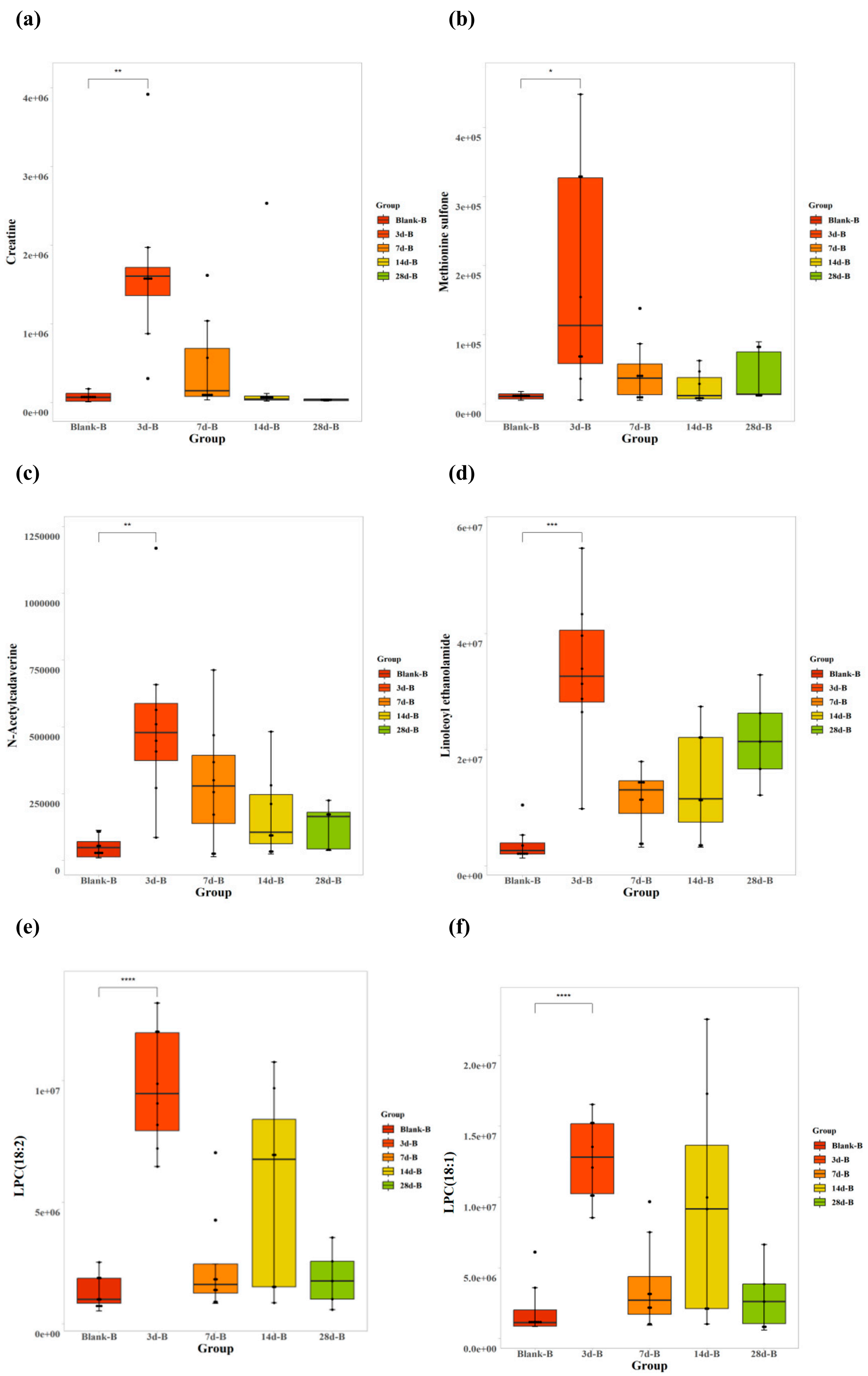

(a)
**
Creatine
4e+06
3e+06
2e+06
1e+06
0e+00
Blank-B
3d-B
7d-B
14d-B
28d-B
Group
Group
Blank-B
3d-B
7d-B
14d-B
28d-B
(b)
*
Methionine sulfone
4e+05
3e+05
2e+05
1e+05
0e+00
(c)
**
N-Acetylcadaverine
1250000
1000000
750000
500000
250000
0
(d)
***
Linoleoyl ethanolamide
6e+07
4e+07
2e+07
0e+00
(e)
****
LPC(18:2)
1e+07
5e+06
0e+00
(f)
****
LPC(18:1)
2.0e+07
1.5e+07
1.0e+07
5.0e+06
0.0e+00

(g)

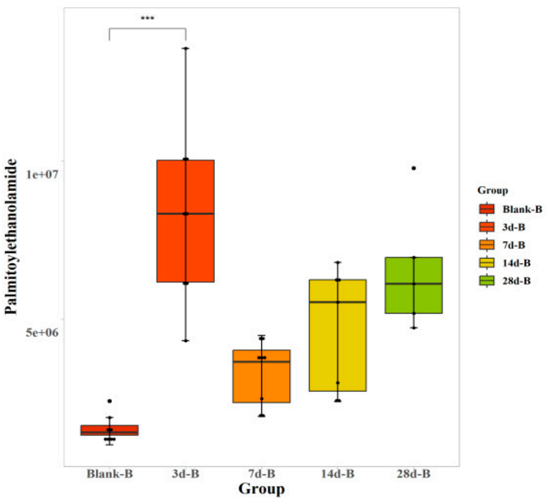
***
Palmitoylethanolamide
1e+07
5e+06
Blank-B
3d-B
7d-B
14d-B
28d-B
Group
Group
Blank-B
3d-B
7d-B
14d-B
28d-B

(h)

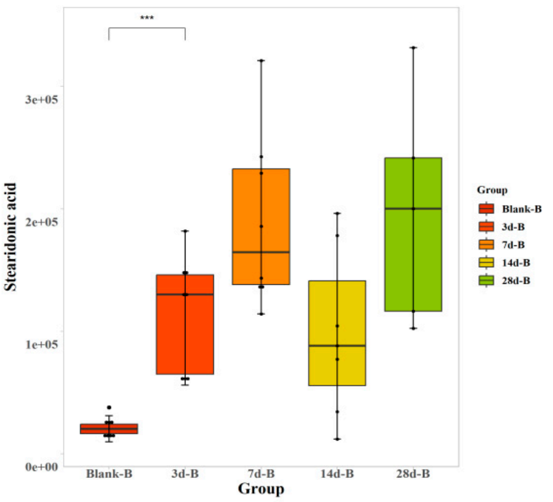
***
Stearidonic acid
3e+05
2e+05
1e+05
0e+00
Blank-B
3d-B
7d-B
14d-B
28d-B
Group
Group
Blank-B
3d-B
7d-B
14d-B
28d-B

(i)

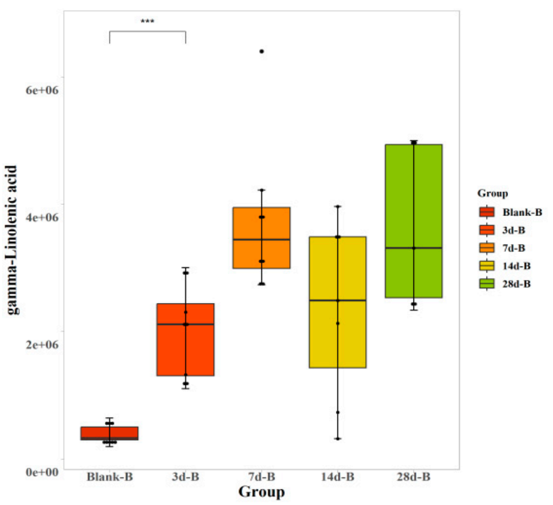
***
gamma-Linolenic acid
6e+06
4e+06
2e+06
0e+00
Blank-B
3d-B
7d-B
14d-B
28d-B
Group
Group
Blank-B
3d-B
7d-B
14d-B
28d-B

(j)

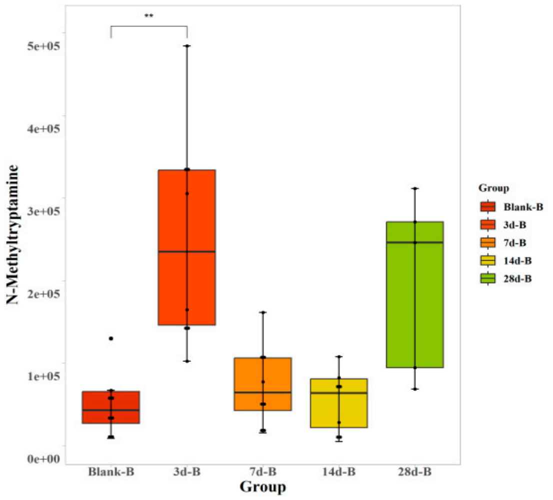
**
N-Methyltryptamine
5e+05
4e+05
3e+05
2e+05
1e+05
0e+00
Blank-B
3d-B
7d-B
14d-B
28d-B
Group
Group
Blank-B
3d-B
7d-B
14d-B
28d-B

(k)

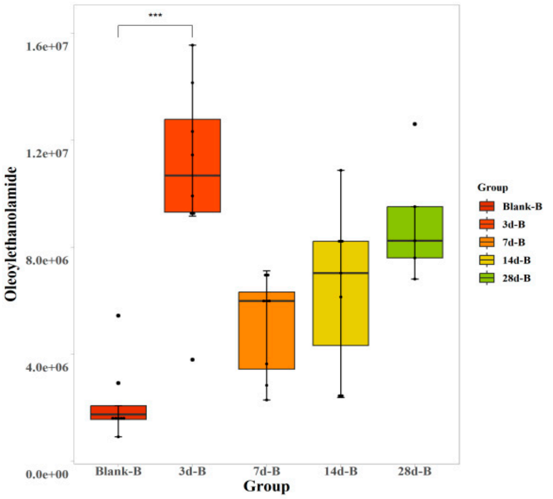
***
Oleoylethanolamide
1.6e+07
1.2e+07
8.0e+06
4.0e+06
0.0e+00
Blank-B
3d-B
7d-B
14d-B
28d-B
Group
Group
Blank-B
3d-B
7d-B
14d-B
28d-B

(l)

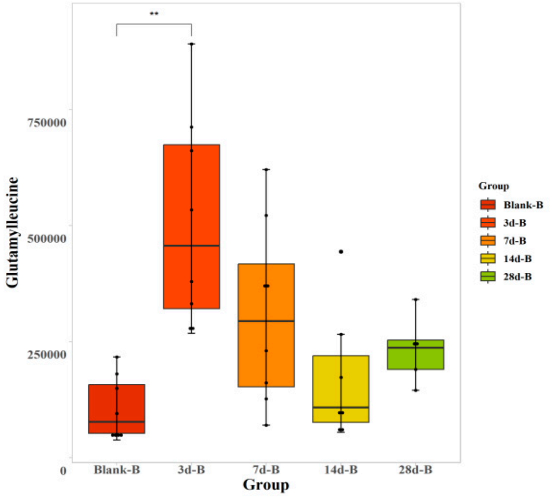
**
Glutamylleucine
750000
500000
250000
0
Blank-B
3d-B
7d-B
14d-B
28d-B
Group
Group
Blank-B
3d-B
7d-B
14d-B
28d-B

(m)

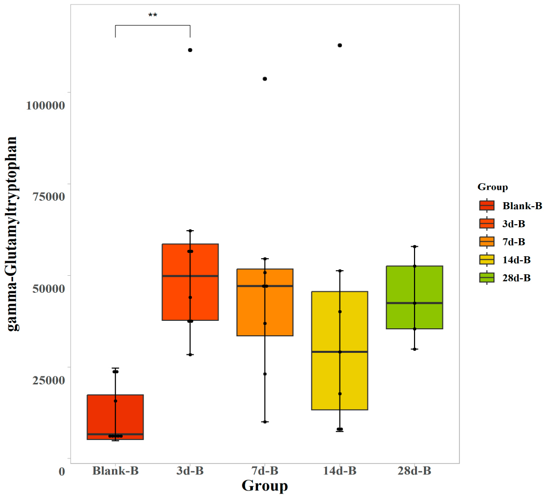

**
gamma-Glutamyltryptophan
100000
75000
50000
25000
0
Blank-B
3d-B
7d-B
14d-B
28d-B
Group
Group
Blank-B
3d-B
7d-B
14d-B
28d-B

(n)

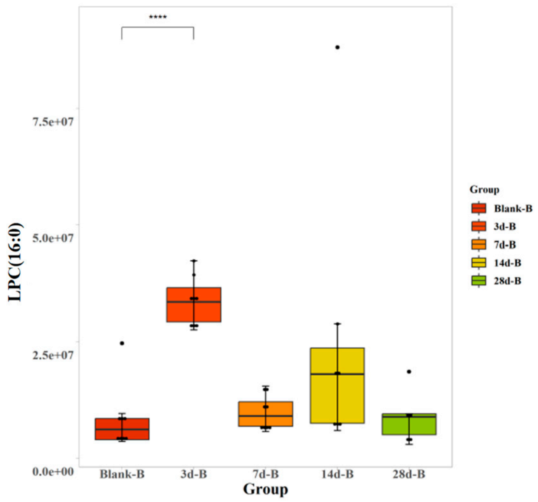

****
LPC(16:0)
7.5e+07
5.0e+07
2.5e+07
0.0e+00
Blank-B
3d-B
7d-B
14d-B
28d-B
Group
Group
Blank-B
3d-B
7d-B
14d-B
28d-B

(o)

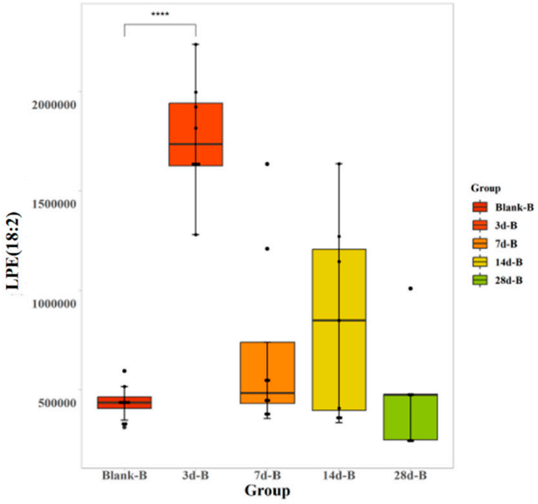

****
LPE(18:2)
2000000
1500000
1000000
500000
Blank-B
3d-B
7d-B
14d-B
28d-B
Group
Group
Blank-B
3d-B
7d-B
14d-B
28d-B

(p)

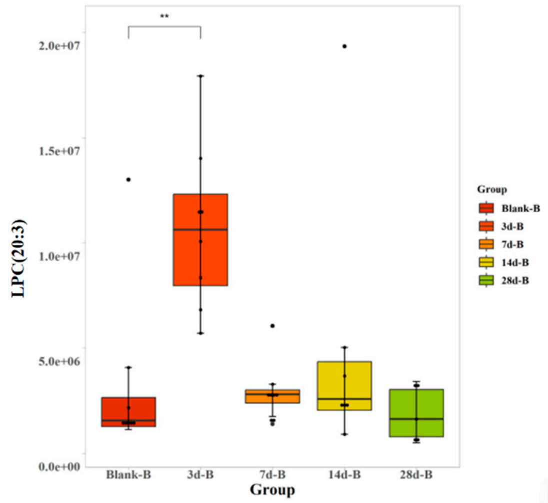

**
LPC(20:3)
2.0e+07
1.5e+07
1.0e+07
5.0e+06
0.0e+00
Blank-B
3d-B
7d-B
14d-B
28d-B
Group
Group
Blank-B
3d-B
7d-B
14d-B
28d-B

(q)

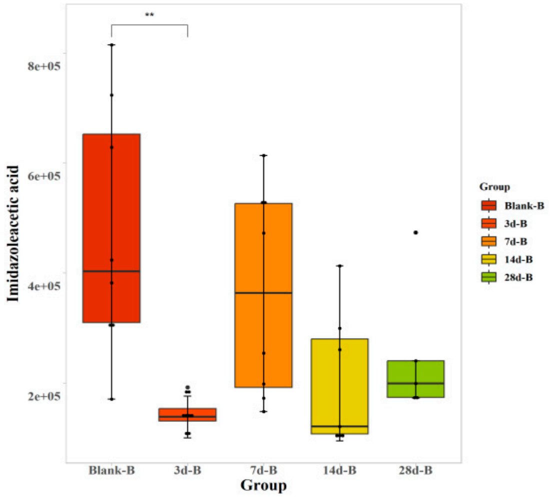

**
Imidazoleacetic acid
8e+05
6e+05
4e+05
2e+05
Blank-B
3d-B
7d-B
14d-B
28d-B
Group
Group
Blank-B
3d-B
7d-B
14d-B
28d-B

(r)

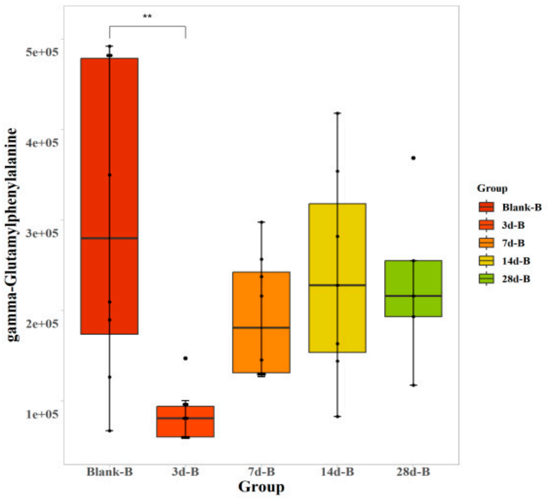

**
gamma-Glutamylphenylalanine
5e+05
4e+05
3e+05
2e+05
1e+05
Blank-B
3d-B
7d-B
14d-B
28d-B
Group
Group
Blank-B
3d-B
7d-B
14d-B
28d-B

(s)

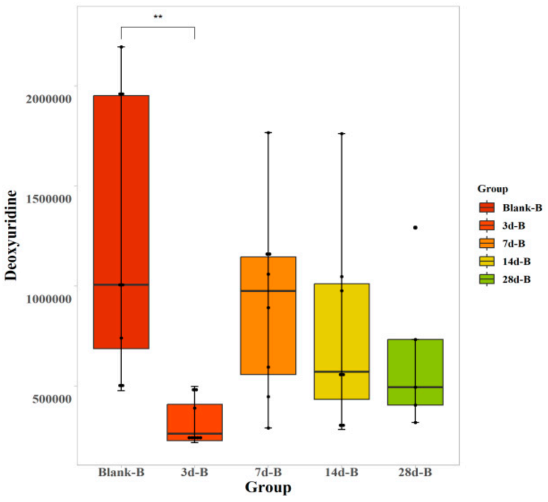

(t)

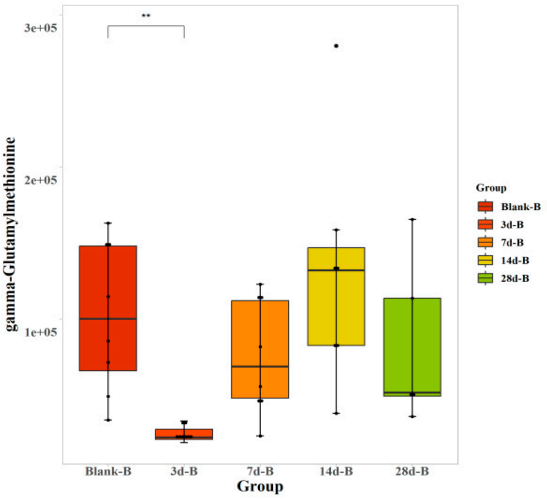

Supplementary Figure S3. Temporal changes of the top 20 compounds ranked by fold change in feces

(a)Creatine; (b)Methionine sulfone; (c)N-acetylcadaverine; (d)Linoleoyl ethanolamide; (e)LPC (18:2); (f)LPC (18:1); (g)Palmitoylethanolamide; (h)Stearidonic acid; (i)gamma-Linolenic acid; (j)N-Methyltryptamine; (k)Oleoylethanolamide; (l)Glutamylleucine; (m)gamma-Glutamyltryptophan; (n)LPC (16:0); (o)LPE (18:2); (p)LPC (20:3); (q)Imidazoleacetic acid; (r) gamma-Glutamylphenylalanine; (s)Deoxyuridine; (t)gamma-Glutamylmethionine;

Radiation group (Group B); Blank-B (pre-radiation in Group B); 3d-B (3 days post-radiation in Group B); 7d-B (7 days post-radiation in Group B); 14d-B (14 days post-radiation in Group B); 28d-B (28 days post-radiation in Group B).

*Statistical significance vs. pre-radiation: *P < 0.05, **P < 0.01, ***P < 0.001.*

(a)

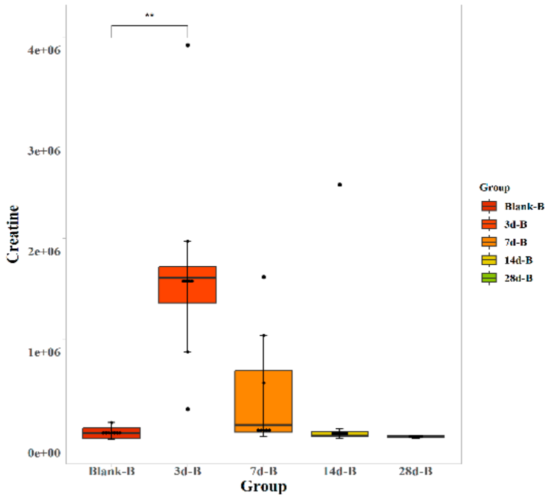
**
4e+06
3e+06
2e+06
1e+06
0e+00
Creatine
Blank-B
3d-B
7d-B
14d-B
28d-B
Group
Group
Blank-B
3d-B
7d-B
14d-B
28d-B

(b)

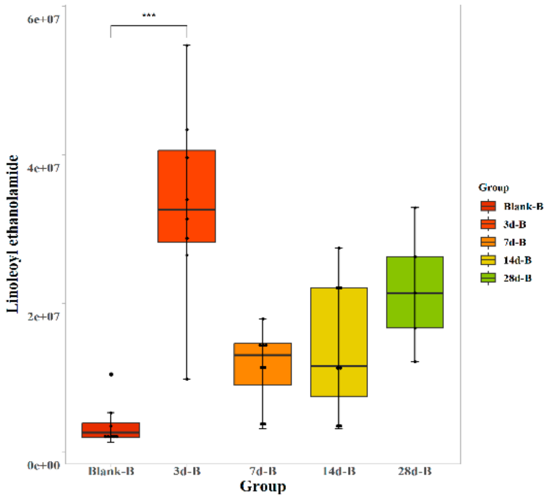
***
6e+07
4e+07
2e+07
0e+00
Linoleoyl ethanolamide
Blank-B
3d-B
7d-B
14d-B
28d-B
Group
Group
Blank-B
3d-B
7d-B
14d-B
28d-B

(c)

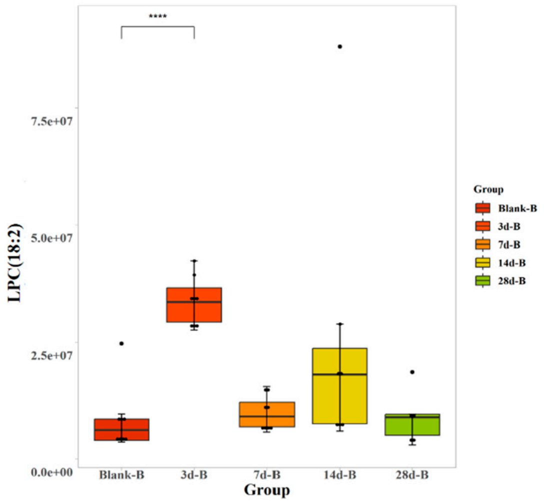
****
7.5e+07
5.0e+07
2.5e+07
0.0e+00
LPC(18:2)
Blank-B
3d-B
7d-B
14d-B
28d-B
Group
Group
Blank-B
3d-B
7d-B
14d-B
28d-B

(d)

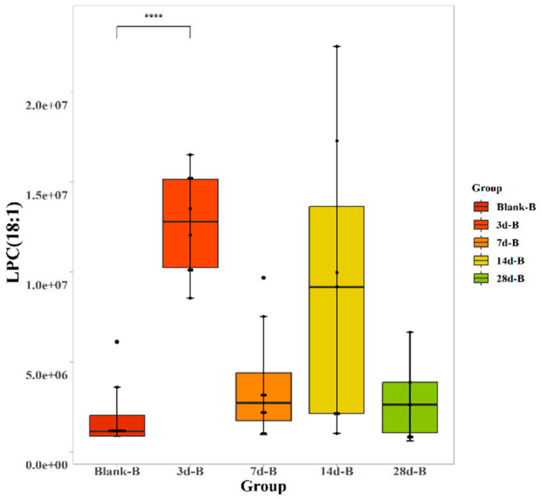
****
2.0e+07
1.5e+07
1.0e+07
5.0e+06
0.0e+00
LPC(18:1)
Blank-B
3d-B
7d-B
14d-B
28d-B
Group
Group
Blank-B
3d-B
7d-B
14d-B
28d-B

(e)

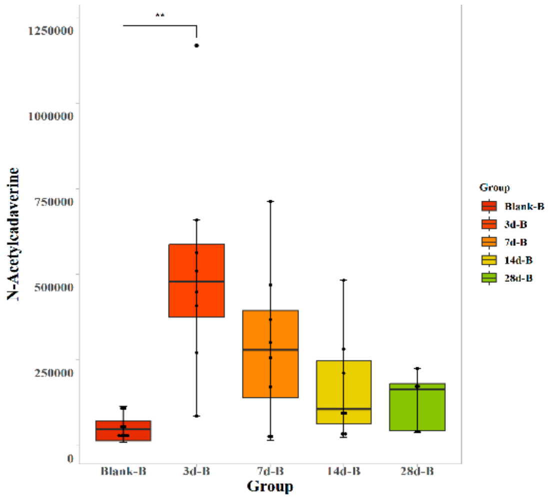
**
1250000
1000000
750000
500000
250000
0
N-Acetylcadaverine
Blank-B
3d-B
7d-B
14d-B
28d-B
Group
Group
Blank-B
3d-B
7d-B
14d-B
28d-B

(f)

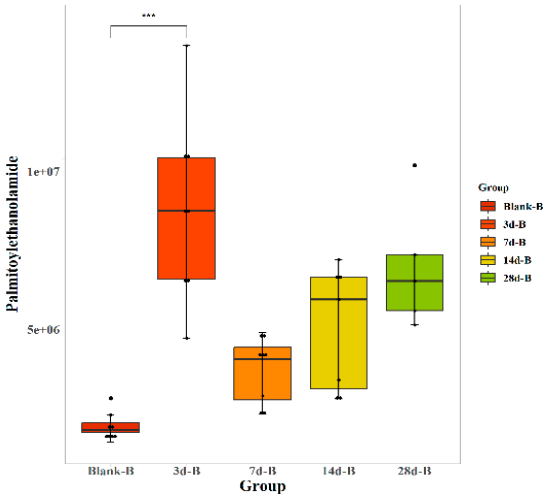
***
1e+07
5e+06
Palmitoylethanolamide
Blank-B
3d-B
7d-B
14d-B
28d-B
Group
Group
Blank-B
3d-B
7d-B
14d-B
28d-B

(g)

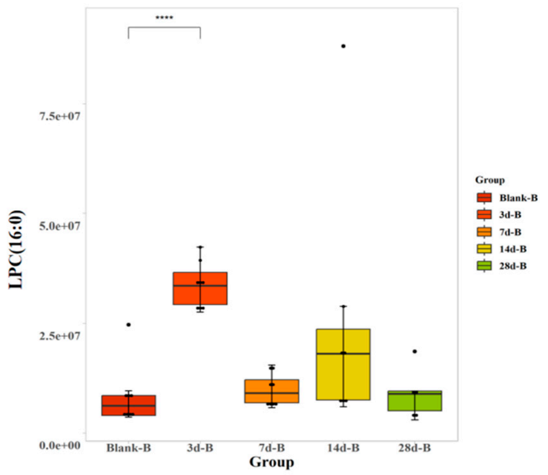
****
7.5e+07
5.0e+07
2.5e+07
0.0e+00
LPC(16:0)
Blank-B
3d-B
7d-B
14d-B
28d-B
Group
Group
Blank-B
3d-B
7d-B
14d-B
28d-B

(h)

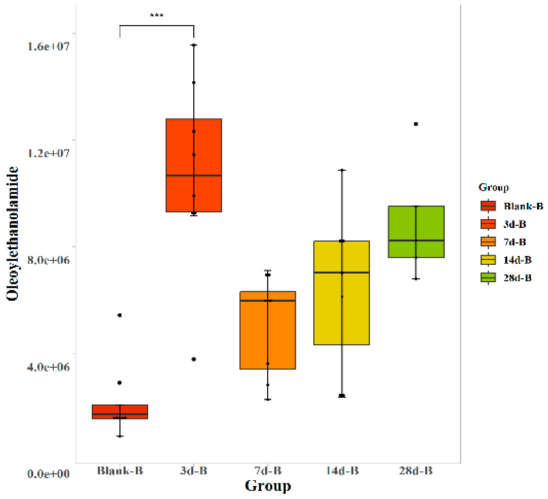
***
1.6e+07
1.2e+07
8.0e+06
4.0e+06
0.0e+00
Oleoylethanolamide
Blank-B
3d-B
7d-B
14d-B
28d-B
Group
Group
Blank-B
3d-B
7d-B
14d-B
28d-B

(i)

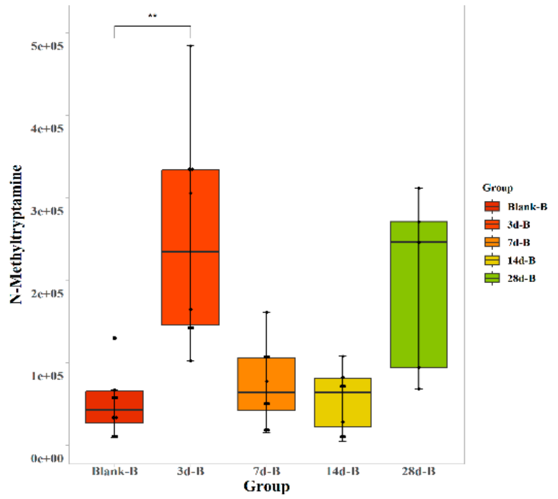
**
5e+05
4e+05
3e+05
2e+05
1e+05
0e+00
N-Methyltryptamine
Blank-B
3d-B
7d-B
14d-B
28d-B
Group
Group
Blank-B
3d-B
7d-B
14d-B
28d-B

(j)

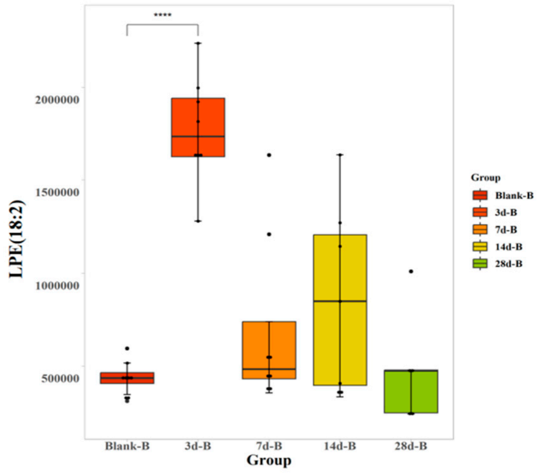
****
2000000
1500000
1000000
500000
LPE(18:2)
Blank-B
3d-B
7d-B
14d-B
28d-B
Group
Group
Blank-B
3d-B
7d-B
14d-B
28d-B

(k)

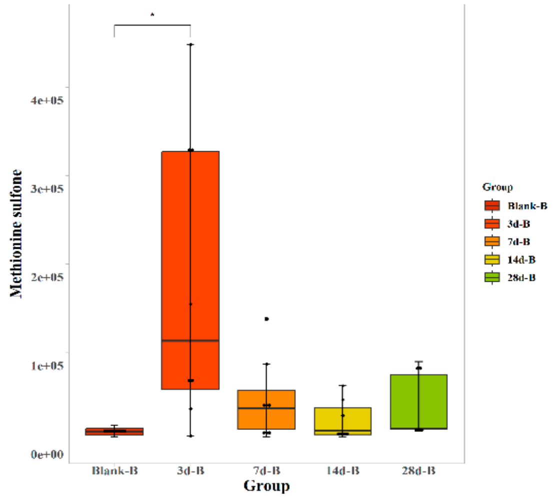
*
4e+05
3e+05
2e+05
1e+05
0e+00
Methionine sulfone
Blank-B
3d-B
7d-B
14d-B
28d-B
Group
Group
Blank-B
3d-B
7d-B
14d-B
28d-B

(l)

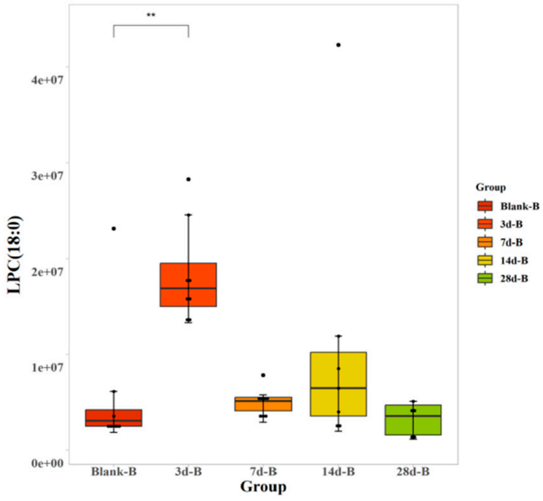
**
4e+07
3e+07
2e+07
1e+07
0e+00
LPC(18:0)
Blank-B
3d-B
7d-B
14d-B
28d-B
Group
Group
Blank-B
3d-B
7d-B
14d-B
28d-B

(m)

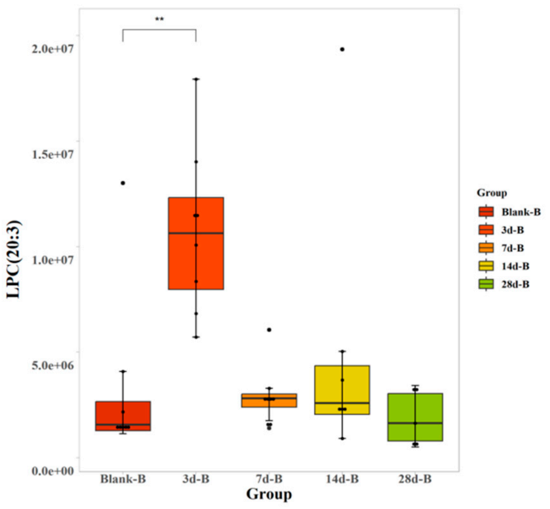
**
LPC(20:3)
2.0e+07
1.5e+07
1.0e+07
5.0e+06
0.0e+00
Blank-B
3d-B
7d-B
14d-B
28d-B
Group
Group
Blank-B
3d-B
7d-B
14d-B
28d-B

(n)

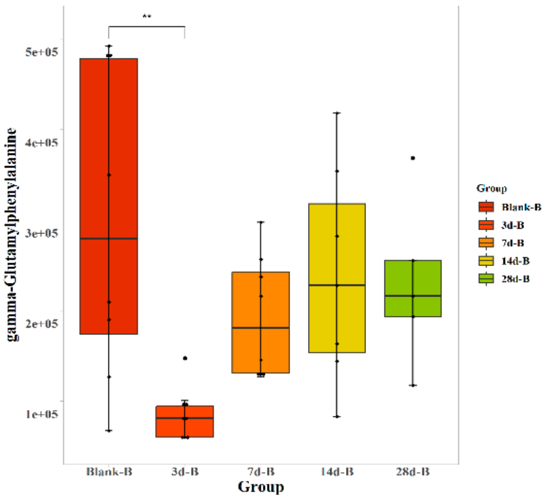
**
gamma-Glutamylphenylalanine
5e+05
4e+05
3e+05
2e+05
1e+05
Blank-B
3d-B
7d-B
14d-B
28d-B
Group
Group
Blank-B
3d-B
7d-B
14d-B
28d-B

(o)

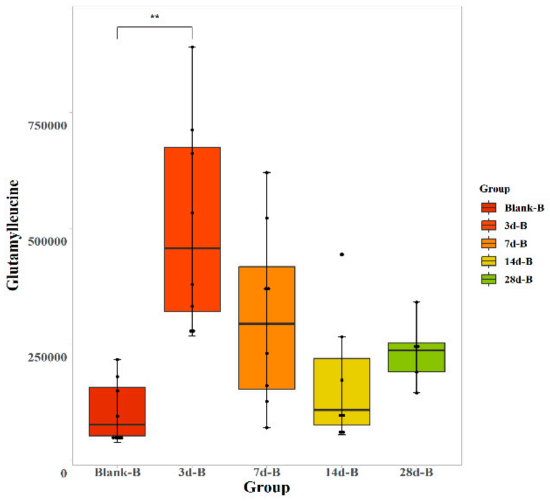
**
Glutamylleucine
750000
500000
250000
0
Blank-B
3d-B
7d-B
14d-B
28d-B
Group
Group
Blank-B
3d-B
7d-B
14d-B
28d-B

(p)

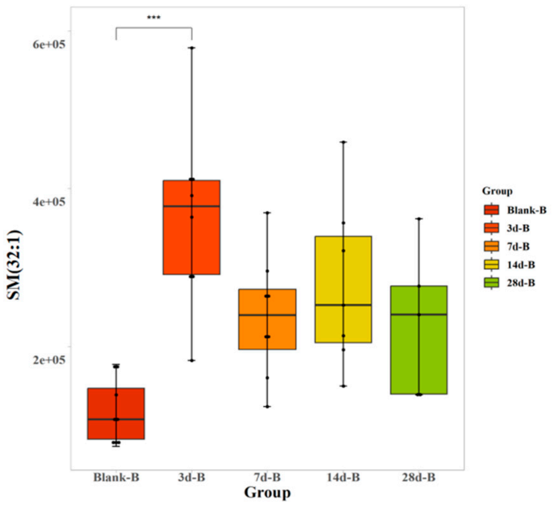
***
SM(32:1)
6e+05
4e+05
2e+05
Blank-B
3d-B
7d-B
14d-B
28d-B
Group
Group
Blank-B
3d-B
7d-B
14d-B
28d-B

(q)

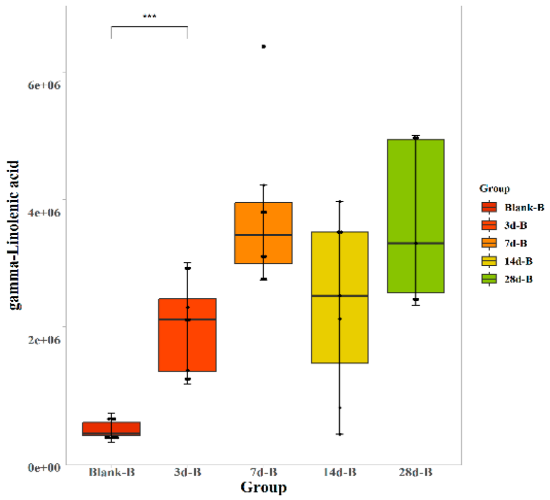
***
gamma-Linolenic acid
6e+06
4e+06
2e+06
0e+00
Blank-B
3d-B
7d-B
14d-B
28d-B
Group
Group
Blank-B
3d-B
7d-B
14d-B
28d-B

(r)

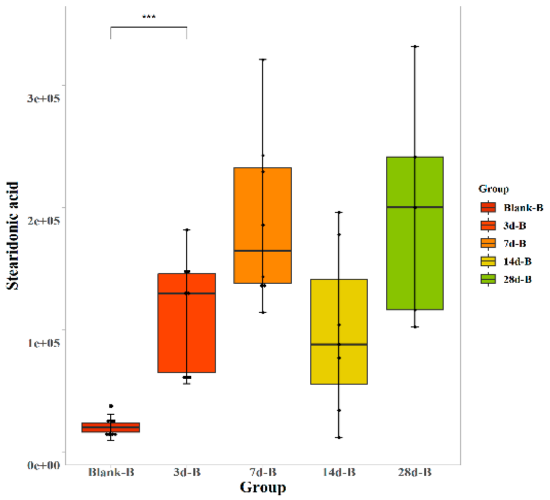
***
Stearidonic acid
3e+05
2e+05
1e+05
0e+00
Blank-B
3d-B
7d-B
14d-B
28d-B
Group
Group
Blank-B
3d-B
7d-B
14d-B
28d-B

(s) (t)

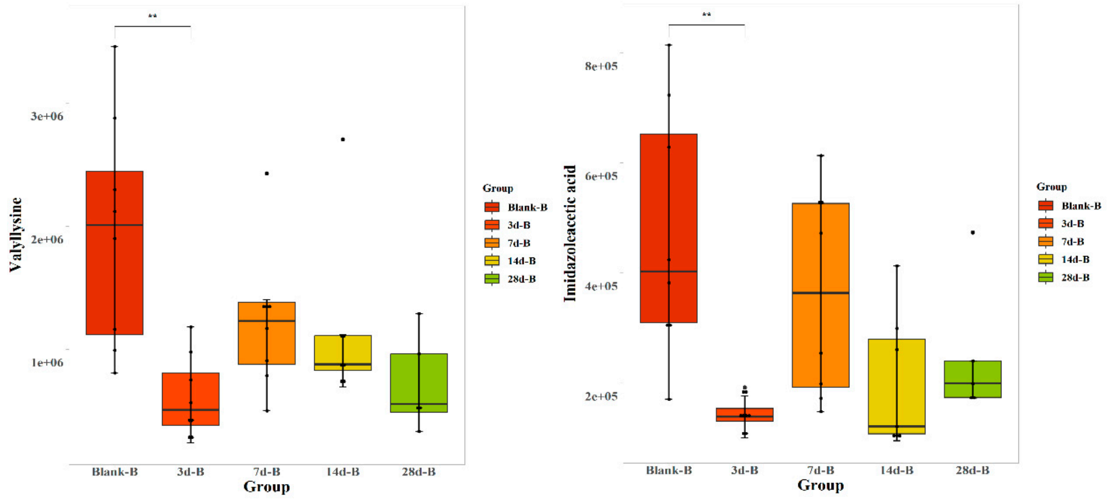

Supplementary Figure S4. Temporal changes of the top 20 compounds ranked by VIP values in the radiation group in feces

(a) Creatine; (b)Linoloy lethanolamide; (c)LPC(18:2); (d)LPC(18:1); (e)N-Acetylcadaverine; (f)Palmitoylethanolamide; (g)LPC(16:0); (h)Oleoylethanolamide; (i)N-Methyltryptamine; (j)LPE(18:2); (k)Methioninesulfone; (l)LPC(18:0); (m)LPC(20:3); (n)gamma-Glutamyltryptophan; (o)Glutamylleucine; (p)SM(32:1); (q)gamma-Linolenic acid; (r)Stearidonic acid; (s)Valyllysine; (t)Imidazoleacetic acid

Radiation group (Group B); Blank-B (pre-radiation in Group B); 3d-B (3 days post-radiation in Group B); 7d-B (7 days post-radiation in Group B); 14d-B (14 days post-radiation in Group B); 28d-B (28 days post-radiation in Group B).

*Statistical significance vs. pre-radiation: *P < 0.05, **P < 0.01, ***P < 0.001.*

(a)

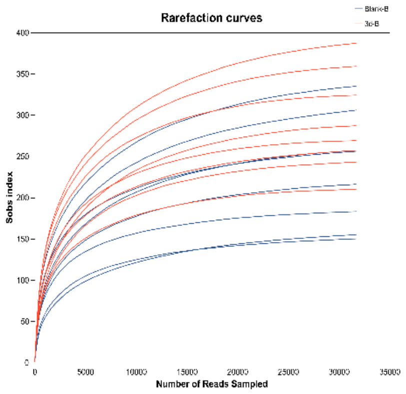

(b)

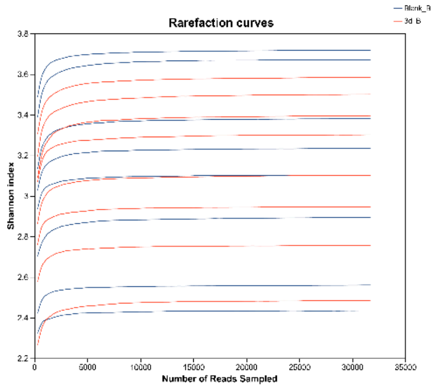

Supplementary Figure S5. Rarefaction curves

(a) Rarefaction curves of the ASV level Sobs index; (b) Rarefaction curves of the ASV level Simpson index.
